# Dual frequency ultrasonic liquid phase exfoliation method for the production of few layer graphene in green solvents

**DOI:** 10.1016/j.ultsonch.2024.106954

**Published:** 2024-06-14

**Authors:** Amanpreet Kaur, Justin A. Morton, Anastasia V. Tyurnina, Abhinav Priyadarshi, Morteza Ghorbani, Jiawei Mi, Kyriakos Porfyrakis, Dmitry G. Eskin, Iakovos Tzanakis

**Affiliations:** a Faculty of Technology, Design and Environment, Oxford Brookes University, Headington, Oxford, OX3 0BP Wheatley, Oxford, OX33 1HX, UK; b Department of Engineering Science, University of Oxford, Parks Road, Oxford, OX1 3PJ, UK; c Brunel Centre for Advanced Solidification Technology, Brunel University London, Kingston Lane, London, UB8 3PH, UK; d Faculty of Engineering and Natural Science, Sabanci University, 34956 Tuzla, Istanbul, Turkey; e Department of Engineering, University of Hull, Cottingham Rd, Hull, HU6 7RX, UK; f Faculty of Engineering and Science, University of Greenwich, Central Avenue, Chatham Maritime, Kent, ME4 4TB, UK; g Department of Materials, University of Oxford, Parks Road, Oxford, OX1 3PH, UK

**Keywords:** Few-layer graphene, Acoustic pressure, Ultrasonic exfoliation, Eco-friendly solution, Shock wave emission

## Abstract

In this work, we implement a dual frequency (24 kHz and 1174 kHz) ultrasonic assisted liquid phase exfoliation (ULPE) technique in deionized water (DIW) and other eco-friendly solvents, to produce a variety of high-quality few-layer graphene (FLG) solutions under controlled ultrasonication conditions. The resulting FLG dispersions of variable sizes (∼0.2–1.5 μm^2^) confirmed by characterisation techniques comprising UV–Vis spectroscopy, Raman spectroscopy and high-resolution transmission electron microscopy (HR-TEM). For the first time we demonstrate that high yield of FLG flakes with minimal defects, stable for 6 + months in a solution (stability ∼ 70 %), can be obtained in less than 1-hour of treatment in either water/ethanol (DIW:EtOH) or water/isopropyl alcohol (DIW:IPA) eco-friendly mixtures.

We also scrutinized the underlying mechanisms of cavitation using high-speed imaging synchronized with acoustic pressure measurements. The addition of ethanol or IPA to deionized water is proposed to play a central role in exfoliation as it regulates the extend of the cavitation zone, the intensity of the ultrasonic field and, thus, the cavitation effectiveness. Our study revealed that lateral sizes of the obtained FLG depend on the choice of exfoliating media and the diameter of a sonotrode used. This variability offers flexibility in producing FLG of different sizes, applicable in a wide spectrum of size-specific applications.

## Highlights

• Equal volume ratio mixtures of Water-Ethanol and Water-isopropyl alcohol eco-friendly solvents are found to be processing efficient for the production of stable few layer graphene.
• Water-Ethanol and Water-isopropyl alcohol mixed solvents have low cavitation pressure threshold.
• Sonotrode surface area is an important factor in affecting the cavitation zone and exfoliation processing times.
• High quality graphene of good yield (6 %), with tailored thicknesses (4–10 layers), stable for 6 + months in less than 1-hour of treatment is produced.

## Graphical abstract

**Figure:**
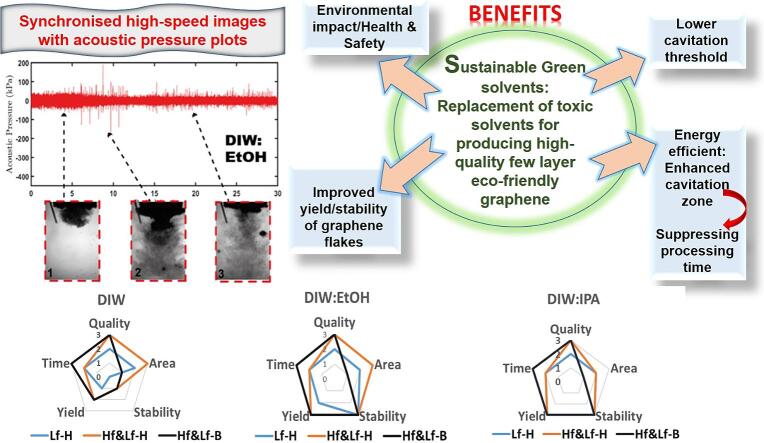

## 1 Introduction

To date, there is plethora of methodologies available in the literature for producing graphene, specifically micro-mechanical exfoliation [1], [2], chemical vapour deposition (CVD) growth of graphene[2] and chemical oxidation of graphite [4]. However, these methods have bottlenecks in terms of yield and throughput to be scaled-up due to their inefficiency or poor control of layers. Alternative route, *i.e.* ultrasound-assisted liquid phase exfoliation (ULPE) has been recognized to be highly promising due to being facile, cost-effective, potentially up-scalable and versatile, in which final liquid dispersions can be further employed to deposit on different substrates in a variety of environments [4]. In order to harness the phenomenal properties of graphene in high-end applications such as inkjet printing [5], conductive inks [6], [7], thermal management pastes [8], anti-corrosion coatings [9] etc., its stable dispersion either in aqueous or environmentally benign liquid media is a pressing requirement to the final applications.

To achieve this, the key strategy here is in the selection of potential solvents or mixture of solvents either binary or ternary, which can efficiently exfoliate and stabilize graphene. This can be achieved using controlled ultra sonication conditions and when the surface energy of solvent closely aligns with graphene (∼68 mJ/m^2^) [1]. By some means, if the yield/stability can be improved in a properly selected solvent or solvent-mixtures under given experimental ULPE conditions, then the chosen solvent-based exfoliation method will be greatly preferred over harsh chemical-based methods that are currently most widely used, *e.g.*, Hummers method [3], Lithium intercalation [11] etc. This is especially important for bio-medical applications such as drug delivery, where graphene needs to be of high purity and biocompatible. Besides, a significant amount of ULPE produced graphene can be further exploited to develop low-temperature processed electrically conductive graphene-based pastes and inks replacing metal-based electrodes to be used in perovskite solar cells and temperature-sensitive semiconductors [12].

Currently, the majority of LPE graphene is produced using 1-methyl-2-pyrrolidone (NMP) or dimethylformamide (DMF) solvents due to their effectiveness in chemically exfoliating graphite and generating stable graphene dispersions [13]. However, their toxicity and difficulty in removal from the final exfoliated product due to their high boiling points present challenges in terms of eco-friendly and sustainable graphene manufacturing.^12^ Moreover, they are classified as carcinogenic and reproductive toxins by environmental, health and safety considerations (EHS) [13]. Therefore, industrial scale use of such solvents may pose severe health issues and environmental risks. Besides, it needs dangerous goods shipping certifications, which adds to the expense and creates complications when it has to be transported. Furthermore, health and safety rules require the use of expensive equipment such as fume hoods and exhausts, which has a direct impact on production costs. In view of the toxicity of solvents and scale-up of process, identification of green solvents for ULPE is timely and necessary.

Our group has demonstrated that ethanol and isopropanol can exfoliate graphite as effectively as NMP [15]. Recently, mixed-solvent strategy has been introduced in which two or more eco-friendly solvents are synergized to produce strong hydroalcoholic co-solvents for direct ULPE of 2D materials [16]. The positives of using co-solvents to that of single solvent are: (1) it avoids the use of toxic solvents (as previously mentioned) by designing a new green co-solvent with similar surface tension properties to the single toxic one; (2) it optimizes the ULPE process of different 2D materials by simply altering co-solvent volume ratios; (3) It prevents aggregation during the solvent evaporation process, which is a case of organic solvents with high boiling points; (4) it reduces cost and production complexity; and (5) it improves material's dispersibility, whereas similar neat solvents have little or no solubility [17].

Recently, Hernandez and co-workers [10] have demonstrated the criterion on the basis of Hildebrand solubility parameters, Hansen solubility parameters and surface tensions to compile a list of selected solvents which are efficient in dispersing graphene. Their findings indicate that for efficient solvents, it is essential to have surface tensions that correspond to graphene, as this reduces the enthalpic cost of mixing. In addition to improve graphene dispersibility/wettability in a given solvent, the other crucial requirement for a solvent is its ability to stabilize suspended graphene. The interactions between graphene and the solvent must possess enough strength to counterbalance the strong van der Waals (vdW) attractive forces exerted by the graphene sheets [4]. As a result, solvents that tend to remain contained between the graphene sheets rather than in the bulk-solvent phase are considered to be the best solvents for efficiently and steadily dispersing graphene. In this direction, co-solvent approach is often applied by mixing two solvents having dissimilar physical properties in appropriate volumes to regulate the resultant surface tension of the solvent ideal for exfoliation of layered materials [15], [16].

**Fig. 1:**
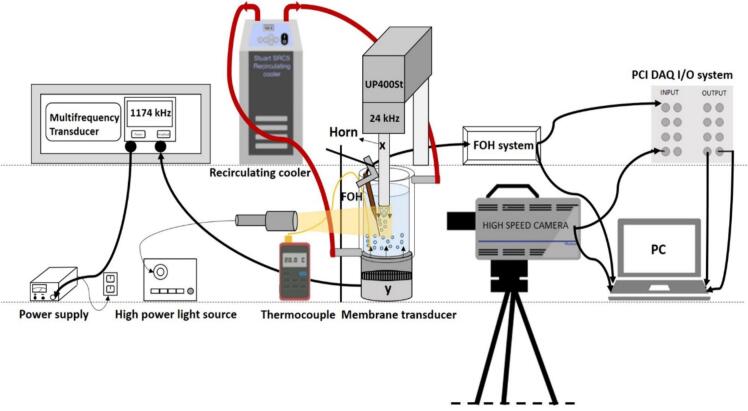
An illustrative diagram showcasing the implementation of acoustic detection equipment in the dual frequency (Hf&Lf) ULPE of graphene.

This work demonstrates the exfoliation role of eco-friendly solvent/co-solvents: deionized water (DIW), deionized water:ethanol (DIW:EtOH) (1:1), and deionized water:isopropyl alcohol (DIW:IPA) (1:1) for producing graphene using a novel dual frequency cavitation reactor with the aid of systematic characterisation studies complemented with acoustic pressure measurements whose schematic is given in Fig. 1. There are several research studies that feature ULPE of graphite focussing the effect of input power [18], [19], sonication duration [20], initial graphite structure [4], single solvent [21] etc. However, these studies do not address the implications of utilizing two different ultrasonic frequencies in conjunction with varying sonotrode sizes on the ultrasonic liquid phase exfoliation of graphene in alcohol-based co-solvents. Our group [20], [23], [26], [37] previously addressed the positives of using high frequency (Hf) and low frequency (Lf) sources where thinner (3–5 layers) and large sized graphene flakes (∼1 μm^2^) were produced in DIW. However, low yield and stability issues always remain bottleneck for dispersing graphene in DIW [20]. Therefore, in this study, we take the next step by introducing the previously identified green solvents for efficient exfoliation [14], [22], [31] such as DIW:EtOH and DIW:IPA plus a range of ultrasonication conditions including two different shape-type sonotrodes with different surface area. Experiments were conducted under synchronised acoustic pressure measurements with high-speed imaging which allows us to optimise the parameters and look deeper into the role of solvents in the cavitation induced exfoliation mechanisms. Results demonstrate that the mixture of the green solvents within this novel dual frequency reactor set up produce high-quality FLG flakes (up to 1.5 μm^2^) with good yield (∼6%) and high throughput, achieving the desired quantity in less than 1 h while maintaining stable suspensions for over 6 months (stability ∼ 70 %).

## 2 Methods

### 2.1 Materials

This initial step involves the identification and selection of specific materials essential for the experiment as indicated in Table 1 below.

**Table 1:** List of required materials for experiments.

Material	Specification	Company
Graphite powder (GP)	300 mesh, particle size 56 μm	Alfa Aesar, UK
De-ionized water (DIW)	Ultra-Pure	Hexeal Chemicals, UK
Ethanol (EtOH)	99.9 %, ultra-pure	Merck Life Sciences, UK
Isopropyl alcohol (IPA)	99.9 %, ultra-pure	Merck Life Sciences, UK
Silicon wafers	Diameter 7.6 cm, Orientation 〈1 0 0〉	Pi-Kem, UK
Holey carbon coated copper grid	300 mesh	Agar Scientific, UK
Acetone	99.9 %, ultra-pure	Merck Life Sciences, UK
Polytetrafluoroethylene (PTFE) membranes	0.2 µm pore size, 47 mm in diameter	Merck Millipore, UK

### 2.2 Experimental set-up

Fig. 1 shows the schematic diagram of our dual-frequency ULPE system. The ULPE experiments were carried out in a double-jacketed beaker (Cole Parmer, 50-mm diameter) filled with DIW (150 ml), DIW:EtOH (1:1) (DIW (75 ml) + EtOH (75 ml)) and DIW:IPA (1:1) (DIW (75 ml) + IPA (75 ml)). The rationale behind this specific choice of 50:50 ratio of DIW:EtOH/DIW:IPA is due to its surface tension (23.91 mN/m) being close to the surface energy of graphite (23.93 mN/m), that promotes wettability and dispersibility which provides more appropriate physical properties beneficial for high exfoliation efficiency of graphene as previously reported by our group [14], [22], [31]. A titanium horn (H) sonotrode with a diameter of 22 mm (marked as x in the schematic shown in Fig. 1) attached to a Heilscher UP400St piezoelectric transducer operating at a frequency of 24 kHz (identified as low frequency Lf-H) was immersed 10 mm below the liquid surface. A bell-shaped (B) sonotrode fixed to similar Lf transducer (designated as Lf-B) of 40 mm diameter was also used for ULPE experiments. The another ultrasonic (US) source of 1174 kHz (designated as high frequency, Hf) was operated from the bottom of the beaker (marked as y in the schematic) using a multi-frequency membrane transducer (Meinhardt Ultrasonics) with a Ti diaphragm (50 mm in diameter). The beaker was attached to a recirculating chiller (Cole Parmer Stuart SRC5) through hose pipes providing for temperature control. Once the desired temperature of 40 ± 2 °C was reached (monitored with an RS 52 digital thermometer) based on our previous sono-exfoliation studies protocols [24], pre-weighed GP (60 mg) was added to the studied solvents (DIW, DIW:EtOH, and DIW:IPA) and stirred in manually for homogeneous dispersion. Continuous sonication was performed for 2-h with the horn-type (cylindrical) sonotrode while the duration of sonication for the bell shaped sonotrode was 1-h. It is to be noted that we had performed the series of optimisation experiments with H/B sonotrodes that enabled us to choose the specific sonication time durations of 1 and 2 h based on their size and input power for the final set of experiments and results are provided in Supplementary ((Figure S1-6, Figure S8, Figure S10)). Both transducers were operated at 50 % of their maximum input power (The optimization experiments for using 50 % power are given in Figure S1-4). The maximum input power setting of Lf transducer is 100 % with peak-to-peak amplitude 46 μm and 18 μm, operating with H and B sonotrodes, respectively. However, the maximum input power (in W) differs from liquid to liquid and is estimated in Table S1 along with acoustic intensity and sonication energy delivered during ULPE.

### 2.3 Characterisation

Subsequently, collected dispersions after ULPE were centrifuged at 1500 *g* (The relative centrifugal force (RCF) is calculated in gravity (*g*) units, specifically as the g-force in m/s^2^) for 15 min using a SciSpin One Benchtop centrifuge to separate un-exfoliated graphite particles/thick flakes and retrieve supernatants. The process optimisation parameters for centrifugation speeds can be found in our previous work^22^ Finally, attained supernatants were designated as Hf&Lf-DIW:IPA (H), Hf&Lf-DIW:EtOH (H), Hf&Lf-DIW (H), Hf&Lf-DIW:IPA (B), Hf&Lf-DIW:EtOH (B) and Hf&Lf-DIW (B), representing samples were processed in DIW:IPA, DIW:EtOH, DIW respectively in dual-frequency (Hf & Lf) configuration using H and B sonotrodes.

The UV–Vis absorption spectra of the freshly acquired supernatants were then scanned in the wavelength range of 200–800 nm using (Cary-60 spectrophotometer, Agilent Technologies) quartz cuvettes (volume 3.5 ml, optical path length 10 mm, Agilent Technologies). The measurements were repeated three times to ensure the consistency in results. After detecting graphene peaks in the UV–Vis spectra, the scanned supernatants were drop-cast onto a cleaned silicon substrate and dried in a vacuum oven. The drop-cast samples were then micro-Raman analyzed using an InVia spectrometer (Renishaw) with a 514 nm laser excitation wavelength (2.33 eV). To avoid damage to the sample and any potential shifts in the peak, the laser power was maintained below 1 mW. Raman spectra of 20–30 random graphene flakes were registered with a 50 × magnification in the range from 1200 to 3100 cm^−1^ and consistent signal-to-noise ratio was achieved by adjusting the acquisition time appropriately. Concurrently, 2–3 supernatant drops were applied to a holey carbon-coated copper grid and then dried completely for transmission electron microscopy (TEM) observations. A JEOL 2100F Field Emission Gun running at 200 kV was used to conduct TEM investigations (both low and high resolution) on individual 30–45 graphene flakes to determine their area and number of layers (NLs). To generate meaningful statistical data, image processing was performed using *ImageJ* software for thickness and surface area estimations of each interrogated flake. Consequently, the top part of the supernatants for each case (approx. 100 ml) was vacuum filtered on 0.2 µm pore PTFE membrane and dried completely. The membrane was weighed before and after filtration to determine the mass difference for yield estimations, as explained in [22].

### 2.4 Acoustic pressure measurements

Acoustic pressures were measured for each solvent using both the H and B sonotrodes along with the high frequency membrane transducer within the dual-frequency ULPE reactor. The pressures were measured using a calibrated fibre optic hydrophone (FOH) (Precision Acoustics Ltd) with a calibration range between 1 and 30 MHz in 1 MHz steps, with a linear sensitivity particularly within the 1–5 MHz range indicating the absence of structural resonance in the hydrophone. Detailed sensitivity values for different frequencies can be found elsewhere [24], [35]. This frequency range is appropriate for detecting broadband shock waves (SWs), which have already been established to be the driving mechanism of exfoliation [23], [24]. The maximum-recorded pressure (P_Max_) records the maximum acoustic pressure from each waveform, and then averages it across all waveforms (giving insight into SWs generated by the bubble clouds beneath the sonotrode). Root mean square pressures (P_RMS_) were also calculated, considering all the generated acoustic emissions. A structured process for signal processing, calibration and pressure conversion can be found elsewhere [27].

### 2.5 Synchronized audio-visual experiments

A Peripheral Component Interconnect (PCI) card was installed into the PC unit to enable synchronized high-speed imaging and acoustic pressure measurements (Fig. 1). Using recorded pressure measurements, the experiment's goal was to view the cavitation zone and generated bubble clouds of each liquid in real time. A Photron- SA-Z 2100 K camera was used at a frame rate of 100,000 fps over 256 × 376 pixels, while using a shutter speed of 8.04 μs (Fig. 1). A light beam LED flash lamp (GS Vitec) was positioned by the container to provide illumination while recording. A titanium sonotrode (3-mm diameter, Hielscher UP200S) with working frequency of 24 kHz was set to use a peak-to-peak amplitude of 126 μm for observation of the cavitation zone. Compared to the ULPE experiments, a smaller diameter probe was used because it allowed for the best field of vision to resolve bubble dynamics without obstructing the camera's field of view due to populated bubbly structures, resulting in more clear and correct data for analysis. Further experimental details can be found in our recently published work [23].

## 3 Results and discussion

### 3.1 UV–Vis spectral analysis and yield estimations

**Fig. 2:**
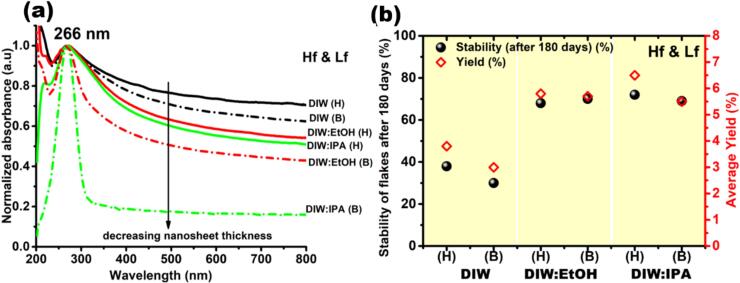
(a) Depicts the normalized UV–Vis absorption spectra of the graphene produced following ULPE (b) yield estimates of the graphene obtained after vacuum filtration (on the right Y-axis) and stability investigations of suspended graphene flakes after 180 days (on the left Y-axis).

Fig. 2 (a) presents the UV–Vis absorption spectra (normalized to maximum peak intensity) recorded for Hf&Lf-DIW:IPA (H), Hf&Lf-DIW:EtOH (H), Hf&Lf-DIW (H), Hf&Lf-DIW:IPA (B), Hf&Lf-DIW:EtOH (B) and Hf&Lf-DIW (B). First thing to note, typical graphene absorption peaks centred at ∼ 266 nm have been observed in each sample, ascribed to π-π* transitions of C = C sp^2^ bonds [25]. The solid and dotted absorption curves present characteristic spectra acquired from samples that were sonicated utilizing H and B sonotrodes respectively. However, we can notice difference in the slopes of the absorption curves between H and B samples. Specifically, samples Hf&Lf-DIW:IPA (B), Hf&Lf-DIW:EtOH (B) and Hf&Lf-DIW (B) determine a stronger thinning effect in flakes, according to [29] (indicated with downward arrow) in contrast to Hf&Lf-DIW:IPA (H), Hf&Lf-DIW:EtOH (H) and Hf&Lf-DIW (H). The bell-sonicated samples show comparatively sharper peaks for each solvent, implying a better dispersion uniformity, an enhanced degree of exfoliation, and an increased likelihood of obtaining few layer graphene (FLG) [25]. After gaining qualitative impression about the exfoliated graphene flakes, it is important to understand the quantitative insights such as the amount of produced graphene (yield) and stability of suspended flakes in each solvent for each ULPE process. The term “yield” (%) refers to ratio of final concentration (C_f_) of filtered graphene obtained after ULPE, followed by centrifugation to the initial concentration of graphite (C_i_) and is a function of centrifugation speed and initial concentration of GP.^22^ The amount of graphene that has sustained in the supernatant over time is referred to as “stability” and has also been used to assess the solvent's ability to stabilize the graphene suspension. This quantity was derived by noting the absorbance of the suspension at 660 nm (as per Lambert-Beer's law [25], then normalized and converted to percentage). Fig. 2(b) shows stability investigations of exfoliated flakes recorded after 180 days (left Y-axis) and the average yield estimations (right Y-axis) of filtered graphene produced in DIW, DIW: EtOH and DIW:IPA for both H and B sonicated samples. By analysing the graph displayed in Fig. 2(b), it can be determined that produced graphene (designated with red diamonds) in DIW:IPA (6.5 % (H), 5.5 % (B)) and DIW:EtOH (5.8 % (H), 5.75 % (B)) and is nearly twice as much as that produced in DIW (3.75 % (H), 3.0 % (B)) (see Figure S11 for exfoliation efficiencies). In this study, yield ∼ 6 % refers to ∼ 0.025 mg/ml of graphene supernatant enriched with FLG, collected at the end of the ULPE process from an initial graphite concentration of 0.4 mg/ml. Furthermore, most research papers use the term ”yield“ qualitatively or statistically, referring to how many FLG flakes were detected in a specific sample out of the total number of flakes scanned in microscopic investigations (TEM, AFM). Accordingly, yield is also sensitive to centrifugation speeds (apart from initial graphite concentration, exfoliation efficiency of solvent), supernatants obtained with lower centrifugation speeds are more likely to contain heavier bulky graphitic material/large sized graphene flakes, which add up the weight of filtered material at the cost of quality trade-off.

This is even more interesting compared to our previous study [22] where graphene produced in single-frequency reactors yielded approximately half (1.25 % and 3.75 % for 2-h Lf-ULPE in DIW and DIW:EtOH respectively) the amount of graphene produced in this study utilising dual-frequency set-ups. It should be noted that the yield obtained in water and the other green solvents can be further increased by up to 3 times when green surfactants such as sodium cholate (SC) and dodecylbenzene sulfonic acid sodium salt (SDBS) are added.^26^ The effectiveness of exfoliation with the B sonotrode can be attributed to its double diameter of 40 mm, which results in an extended cavitation zone evident in Figure S9. This configuration facilitates improved circulation of GP particles within the treatment volume and a significant more generation of shock waves (SWs), which act as the driving force behind the exfoliation process [23]. Additionally, stability (indicated with black spheres) of suspended graphene flakes in DIW:IPA (72 % (H), 69 % (B)), DIW:EtOH (68 % (H), 70 % (B)) and is two times higher in contrast to DIW (38 % (H), 30 % (B)) under 180 days of observation.

Based on the findings from Fig. 2(b), it can be deduced that the performance of B sonotrode in producing/stabilizing graphene in studied solvents is identical to H sonotrode in half ULPE duration, which makes the potent case for energy efficient ULPE process using a bell sonotrode or a large surface sonotrode. The present work claims 1–2 h of sonication as a more energy-efficient method in comparison to operating ULPE lasting for 48–400 h as reported in the existing literature [42], [43], [44].

This hypothesis, which was previously suggested in [23], appears to be validated by the findings presented in this study. Therefore, based on the UV–Vis absorption findings, we conclude that doubling the sonotrode diameter (*i.e.* from 22 to 40 mm) reduces the treatment time by half, *i.e.*, from 2-h to 1-h to obtain the equivalent results. Interestingly, the obtained data suggest that the addition of EtOH/IPA to DIW in equal volumes plays a synergistic role of improving yield and stabilizing the graphene flakes. It is also emphasized that alcohol-water mixtures are not only environmentally preferable solvents; but also graphene supplied in them will remain stable over considerably long periods of time without compromising its quality.

### 3.2 Raman spectral analysis

**Fig. 3:**
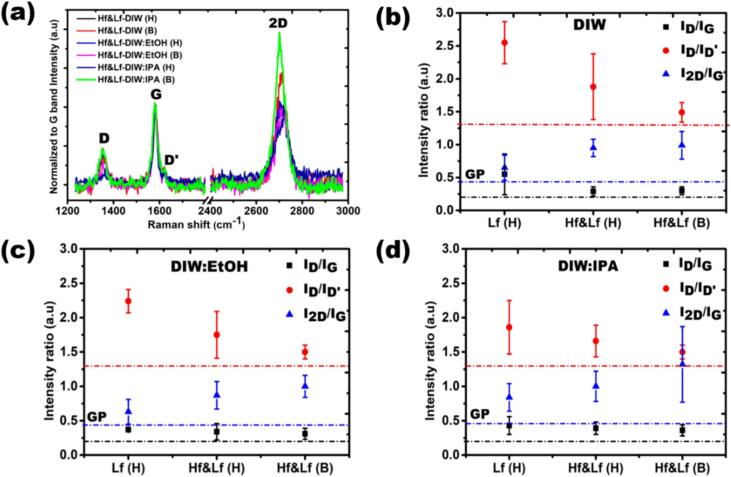
(a) Characteristic Raman spectra of graphene flakes exfoliated in each sample (b)-(d) I_D_/I_G_ (black squares), I_D_/I_D'_ (red circles), and I_2D_/I_G_ (blue triangles) are averaged intensity ratios of registered flakes in DIW, DIW:EtOH, and DIW:IPA, respectively. For reference, the original GP's data is indicated by a dashed line of the same colour. (For interpretation of the references to color in this figure legend, the reader is referred to the web version of this article.)

Based on the obtained UV–Vis results, Raman spectroscopy was further employed to evaluate the defects quantification of the exfoliated flakes and qualitative estimates of their thickness [27]. Fig. 3 (a) exhibits the typical Raman spectra of investigated graphene. The first thing that stands out from each spectrum is the distinctive signature of sp^2^ hybridized carbon, *i.e.* D, G, D′ and 2D bands centred on ∼ 1350 cm^−1^, 1580 cm^−1^, 1620 cm^−1^ and 2720 cm^−1^, respectively [27]. Specifically, the spectra have been normalized to the G-band intensity and adjusted for linear baseline to enable meaningful comparisons. The defect related bands, D and D′ provide information related to the existence of edges, attached functional groups and structural defects in the flakes [28], [29]. We recorded the intensity ratios of the D, D', and 2D bands in relation to the G band for each spectrum. The average values, along with their corresponding error bars, are depicted in Fig. 3(b-d) for DIW, DIW:EtOH, and DIW:IPA, respectively. These plots also contain the data for Lf (H) from our previous work [22] with a single ultrasonic source at 24 kHz to compare the results. The dashed lines of identical color indicate the intensity ratio information of the original GP for comparison. The quality estimates of exfoliated flakes have been evaluated from defect ratios, I_D_/I_G_ (indicated by black squares). From Fig. 3(b-d), we estimated the decline in defect ratios values in interrogated graphene flakes exfoliated in DIW (0.55 to 0.30), DIW:EtOH (0.37 to 0.30) and DIW:IPA (0.43 to 0.36) in Hf&Lf ULPE configuration (second number) in contrast to Lf only (first number). As can be seen from the defect ratios, the Hf-Lf setup produces fewer defective graphene flakes compared to Lf. The observed defect ratios surpass the GP level (I_D_/I_G_ ∼ 0.22, represented by the black dashed line), indicating the emergence of structural defects due to the reduction in thickness of GP crystallites following ULPE [30]. It is worth noting that the defect ratios (I_D_/I_G_) seem to stay consistent for the flakes exfoliated using both H and B sonotrodes. This suggests that the quality of Hf&Lf (H) ULPE-produced graphene flakes over a 2-hour period is comparable to Hf&Lf (B) flakes treated for 1 h. Another interesting feature that can help in evaluating the nature of defects in the exfoliated flakes, is I_D_/I_D′_ (indicated with red circles). The recorded values of I_D_/I_D′_ are in the acceptable range, *i.e.* below 3.5, for each solvent, which makes the case of edge defects rather than basal structural disorders in accordance with Eckmann *et al*. [28], indicative of typical ULPE produced graphene flakes. One can see from Fig. 3 (b-d) that the average values of I_D_/I_D′_ follow the linear decreasing trend from Lf to Hf&Lf for DIW (2.55 to 1.49), DIW:EtOH (2.24 to 1.50) and DIW:IPA (1.86 to 1.50) with the smallest value registered in bell (B) sonicated samples. The recorded values of I_D_/I_D′_ for the registered flakes are higher than those for GP (red dashed line, I_D_/I_D′_ ∼ 1.3), which reflects the variation in orientation of sheets, indicative of ULPE [30].

**Fig. 4:**
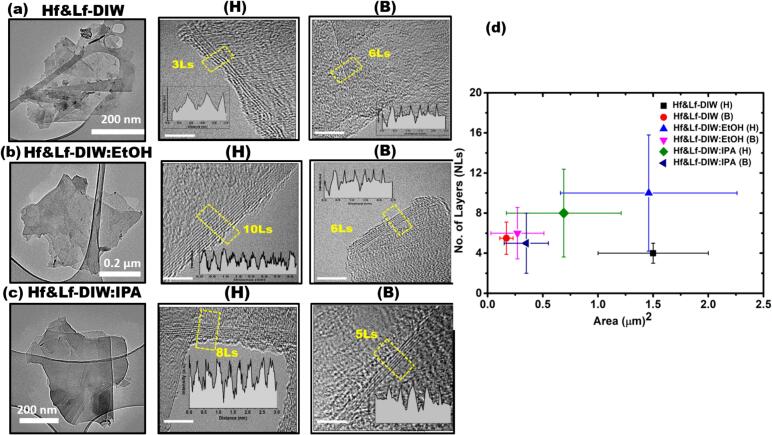
(a)-(c) Typical TEM images of graphene flakes (low and high resolution) (d) Statistical information on the number and area of exfoliated layers for both the H and B sonotrodes in DIW, DIW:EtOH, and DIW:IPA.

It is crucial to examine another significant parameter, which is the ratio of intensity between the 2D and G bands known as I_2D_/I_G_. These values showcase the range in the number of layers and the extent of exfoliation [28]. From Fig. 3 (b-d), the average values of I_2D_/I_G_ (indicated with blue triangle) are increasing from Lf to Hf &Lf for DIW (0.65 to 0.99), DIW:EtOH (0.63 to 0.99) and DIW:IPA (0.84 to 1.32), which corroborate the large population of thin flakes in Hf&Lf processed samples. All the recorded I_2D_/I_G_ ratios for each flake are above the level of GP (blue dashed line, I_2D_/I_G_ ∼ 0.45) which suggests GP crystallites exfoliate to thinner graphene flakes [30], typically FLG (less than 10 layers) with I_2D_/I_G_ ∼ 1 for Hf&Lf configuration [20].

**Fig. 5:**
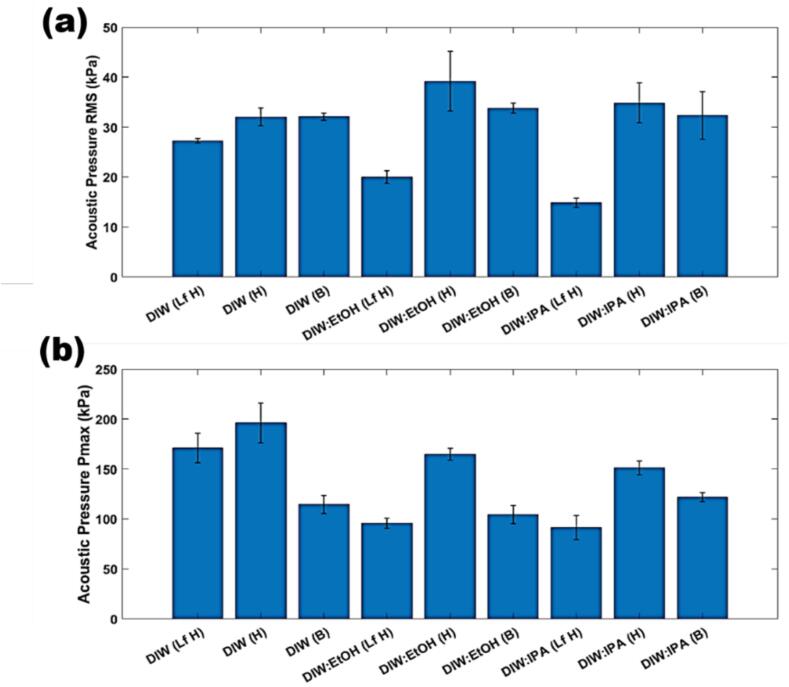
Acoustic pressure measurements in the range of 1–30 MHz from the H and B sonotrode taken for each solvent in-situ just before the end of sonication process (a) P_RMS_ (b) P_Max._. Note that all scenarios for the H and B are in Hf&Lf mode. Lf only is specified with Lf-H.

The generation of exfoliated flakes with fewer defects using the dual-frequency reactor can be credited to the effective combination of different-sized cavitation bubbles and their respective dynamics. For a more comprehensive understanding of the underlying mechanisms, further details can be found in our previous studies [20], [23], [31], [32]. In short, the Lf acoustic emissions result in the formation of larger bubbles or bubbly clouds, measuring a few hundred microns in size, with short lifecycles. These bubbles emit strong shockwaves upon their collapse, effectively loosening the tightly stacked graphite flakes. On the other hand, the Hf source generates smaller bubbles (few microns in size) that vigorously oscillate in a more stable (extended life cycles) manner. These tiny bubbles infiltrate the loose flakes, providing a gentle exfoliation of graphite by operating amidst the initial split and the expanded layers. As a result, the collaboration between these two cavitation regimes demonstrates its benefits for enhancing both the quantity and quality of the exfoliated graphene. It is noteworthy that the values of I_2D_/I_G_ exhibit colonization within a narrow range of 1 ± 0.2, indicating that the majority of FLG is produced in both Hf&Lf (H and B)-DIW and DIW:EtOH (Fig. 3(b-c)). Clearly, there is no substantial variance in I_2D_/I_G_ values for Hf&Lf-DIW(H) and Hf&Lf-DIW(B), which is further discussed on the basis of recorded similar acoustic pressures (Fig. 5a). Conversely, from Fig. 3(d) we notice that I_2D_/I_G_ ratios span a longer range of 1.3 ± 0.6 for Hf&Lf-DIW:IPA(B) samples, identifying single layer graphene (SLG) and bi-layer graphene (BLG) signatures in addition to FLG amongst the registered flakes. However, consistency and absence of significant variations in I_2D_/I_G_ propose the homogeneity of the exfoliated graphene flakes together with the role the physical properties such as surface tension, viscosity and vapour pressure play for cavitation development in investigated hydroalcoholic solutions (see additional details in acoustic section) in agreement with previous studies [33], [34], [35]. The results obtained from Raman spectroscopy are promising, as they indicate that the ULPE Hf&Lf processing technique, utilizing a larger size bell sonotrode, can effectively reduce processing times by half while maintaining the same quality and throughput of few-layer graphene (FLG).

**Fig. 6:**
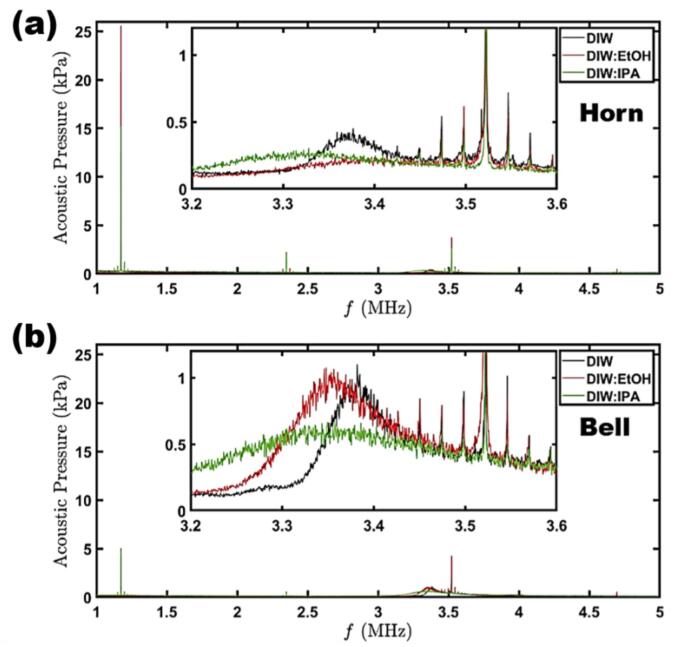
Acoustic spectra of solvents under investigation in the calibration range of the fiber-optic hydrophone. (a) Horn (b) Bell. Note that all scenarios for the H and B are in Hf&Lf mode.

### 3.3 Morphological analysis

To obtain data regarding the exfoliated flakes' dimensions, quality, and number of layers in the investigated solvents, both conventional and high-resolution TEM (HR-TEM) investigations were carried out. Fig. 4 (a-c) demonstrates typical TEM images of graphene flakes exfoliated in DIW, DIW:EtOH and DIW:IPA with both H and B sonotrodes. From the collection of images, we confirm the sheet-like morphology of produced graphene flakes as a consequence of ULPE in each case. The HR-TEM images of flakes exfoliated in each sample reveal the number of layers by edge counting method [36]. The corresponding information is given in the insets of each figure. For every sample, the statistical data for the thickness and area of the flakes under analysis is plotted in Fig. 4d. From graph, it can be seen that the average area of exfoliated flakes in Hf&Lf-DIW:IPA (H), Hf&Lf-DIW:EtOH (H) and Hf&Lf-DIW (H) is 0.7 µm^2^ (olive diamond), 1.46 µm^2^ (blue triangle) and 1.5 µm^2^ (black square) with the average number of layers 8, 10 and 4, respectively. On the other hand, areas of exfoliated graphene flakes in Hf&Lf-DIW:IPA (B), Hf&Lf-DIW:EtOH (B) and Hf&Lf-DIW (B) lie lower with the average areas of 0.35 µm^2^ (navy triangle), 0.27 µm^2^ (pink triangle) and 0.17 µm^2^ (red sphere) with average number of layers 5, 6 and 5 respectively. The bell sonotrode-treated samples have thinner flakes with smaller areas, according to the statistical plot shown in Fig. 4d of the TEM analysis, compared to the horn sonotrode-treated samples. This is understandable since the larger surface of the bell sonotrode promotes more SWs while the larger clouds shield more SWs, resulting in a trade-off that registers similar pressures (see acoustic section). At the same time, the bell sonotrode is more powerful in chopping down and exfoliating the flakes. As a result, size and thickness are better controlled, and quality is comparable, yielding a favourable overall result. Table S3 contains the summarized data for all the investigated parameters (with standard deviation) of the final flakes produced in the studied solvents for both Lf (referenced in our previous studies [21], [22]) and Hf&Lf set-ups. Based on the combined findings from UV–Vis, Raman and TEM data, it is clear that employing the B sonotrode in the same Hf&Lf set-up and liquid environment resulted in an enhanced level of exfoliation in half duration, but produced smaller FLG flakes as a trade-off.

### 3.4 Characterisation of cavitation phenomena in various eco-friendly solvents

#### 3.4.1 Acoustic pressure measurements

Following the characterisation of the produced graphene flakes, further acoustic emissions and visual observations using high-speed imaging were conducted for each ULPE scenario in order to acquire a deep understanding of the role of solvents in the process. P_RMS_ and P_Max_ pressures demonstrated some correlation with the ULPE performance of the solvents (Fig. 5). The Lf pressures were all smaller than their Hf&Lf counterparts, corresponding to the lesser yields produced during ULPE as established in our previous work [22], [37]. Interestingly, the P_RMS_ values for both the H and B sonotrodes were approximately the same (∼30 kPa) (Fig. 5a), which is in-line with the similar measured yields and I_2D_/I_G_ values after Hf&Lf-ULPE (Fig. 2b & 3b-d). Additionally, the P_RMS_ values for DIW in both Hf&Lf cases were less in comparison to the DIW:EtOH and DIW:IPA (Fig. 5a) due to the enhanced cavitation activity driven by the lower surface tension and viscosity of both the solvents (Table S2). Alternatively, DIW (particularly for the H sonotrode with the smaller diameter) generated larger pressures for P_Max_ (primed to detect SW contribution) (Fig. 5b) due to the smaller cavitation zone compared to DIW:EtOH and DIW:IPA, enabling the propagation of a larger number of SWs as a consequence of less shielding as explained in [35].

For DIW:EtOH, the larger measured pressure for the H sonotrode only increased the yield by 0.05 % as compared to the B sonotrode (Fig. 2b). The DIW:IPA solution also produced a marginally higher pressure for the H sonotrode. These similar pressures explain why the B sonotrode can produce comparable graphene yields in half the duration. As the bell (B) has the working surface approximately twice the diameter of the horn (H) sonotrode, circulated GP is more likely to be exposed to powerful SW emissions under the B sonotrode tip. Furthermore, the additional exposure to more SWs directly in the cavitation zone contributes to the smaller FLG cross-sectional areas (Fig. 4d & Table S3). We have previously observed that the emission of SWs mainly arises from the edges of a sonotrode as a result of a collapsing cavitation cloud [24]. As the B sonotrode has a larger periphery, the additionally generated SWs lead to a larger number of interactions with the GP. It is to be noted that despite the larger number of SWs generated *via* the larger diameter of the B sonotrode, the P_Max_ measured is lower due to shielding reducing the maximum pressure of individual SWs.

The P_Max_ pressures were mostly in line with the P_RMS_ measurements (Fig. 5b), however, the case of B configurations featured more suppressed P_Max_ pressures due to shielding of SWs as mentioned above. Lf-only pressure readings were again smaller than the Hf&Lf setups as expected due to lesser cavitation activity. However, DIW produced larger pressures compared to other solvents partly due to the higher propensity for cavitation to collapse, hence, generating powerful SWs unimpeded by shielding (see physical properties such as a higher surface tension Table S2). Although the H sonotrode demonstrated higher pressures than B, we must also consider the size of the vessel and position of the FOH (located at one position only). Due to the larger size of the B sonotrode in comparison to the size of the sonicated environment (vessel size), the edges are close to the vessel walls, hence, most of SWs emitting from the edges may also be obstructed and decay at impact with the nearby solid boundaries. However, despite these reasons explaining why the position of the FOH may pick up lesser pressure readings from the B sonotrode, the smaller sized FLG flakes measured using the TEM (Fig. 4d) indicate the B sonotrode produces enough cavitation and particularly SWs to be effective in breaking up and exfoliating graphite flakes.

Further analysis of the acoustic spectra demonstrated the driving frequency (1.174 MHz, for the Hf source) to generate the highest pressures, followed by the third harmonic (Fig. 6). Interestingly, the majority of the B-sonotrode acoustic pressure is spread out over the 1st and 3rd harmonic (it should be noted this observation is excluding the Lf harmonics as they are outside of the FOH calibration range and therefore filtered out, however, a representative plot in the low frequency range is also presented in Figure S7), with the 2nd harmonic largely suppressed for all the studied solvents. The H-sonotrode gave rise to significantly larger peaks (5 times more for the 1st harmonic), in corroboration with the larger measured pressures and wide distribution of flake sizes (Fig. 6a & 4d). DIW:EtOH demonstrated the most prominent peaks, whereas DIW produced the smallest ones (2.5 times smaller for the H and 2 times smaller for the B at the 1st harmonic), correlating to the FLG yield results discussed comprehensively (Fig. 2b). The reason for this higher-pressure regime in the DIW:EtOH solution has been previously discussed [31] and related to the “mist” of tiny cavitation bubbles that sustain vigorous oscillations, thereby promoting further cavitation activity within the solution. This regime contributes to the exfoliation efficiency as demonstrated in Fig. 4d (larger size flakes similar to the case of DIW although featuring improved stability Fig. 2b). The B-sonotrode spectra peaks were noticeably much smaller in magnitude, indicating strong attenuation of the Hf standing waves as previously reported [31]. This attenuation can be ascribed to the larger diameter of the B-sonotrode that leads to the formation of a larger size cavitation cloud. As the B has double size emitting surface compared to the H-sonotrode, a larger cavitation cloud is formed (Figure S9), hence, obstructing the formation of standing waves while providing a greater contribution to shielding. However, and as expected, this is not the case in the Lf range, where the B-sonotrode exhibits a dominant acoustic signal, reaching up to 40 % (Figure S7).

The hump observed in the insets in Fig. 6 and previously related with the SWs propagation [24] seems to be over twice the magnitude for the B sonotrode as compared to the H sonotrode, indicating a higher SW activity from the larger cavitation clouds under the sonotrode. However, due to cavitation shielding the attenuated SW pressures contributed to an overall lower P_Max_ value than for the H sonotrode (Fig. 5b) as explained previously. Interestingly, the spectra for both the H and the B sonotrodes also produced ultraharmonics around the 1st, 2nd and 3rd harmonics, in intervals of 24 kHz. An example of this can be seen in the insets for both the H and B (Fig. 6) between the ranges 3.45–3.6 MHz, whereby the frequency-domain spikes are separated *via* 24 kHz intervals. This intriguing pattern observed in the MHz range may be attributed to the influence of the fundamental frequency (24 kHz) of the Lf transducer, as previously documented [31].

#### 3.4.2 High-speed imaging synchronised with acoustic measurements

**Fig. 7:**
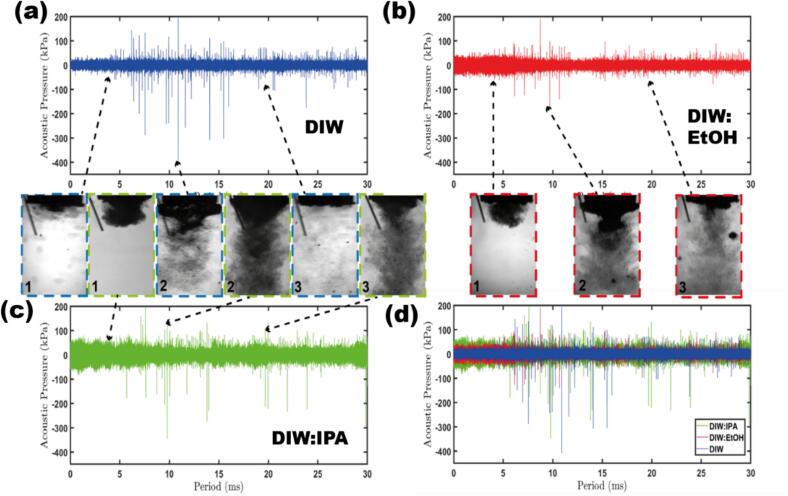
Acoustic pressure plots and synchronized high-speed images (insets) for each liquid under study are shown. (a) DIW (blue); (b) DIW:EtOH (red); (c) DIW:IPA (green); (d) All liquids plotted collectively for comparison. Upon activating the LF source, a 30-millisecond period was recorded. The three representative moments of the developing bubble dynamics for each solvent are shown in the insets. These moments are (1) the initial developing cavitation cloud upon activation of the sonotrode, (2) the maximum cavitation cloud prior to collapse (after which a large corresponding pressure spike is induced), and (3) the stabilised cavitation period. The arrows in the insets indicate these moments. At 100 kfps, imaging was acquired, and the intensity was converted to acoustic pressure using a FOH to measure it. (For interpretation of the references to color in this figure legend, the reader is referred to the web version of this article.)

Fig. 7 presents synchronised high-speed imaging observations with acoustic detection of the studied liquids without the addition of GP. DIW was shown to generate the largest and most frequent SWs (indicated by the sharp individual pressure spikes in the time domain, Fig. 7a), producing a maximum pressure over 400 kPa with the majority peaking around ∼ 200 kPa (in line with the averaged P_Max_ values measured in Fig. 5b). DIW:EtOH and DIW:IPA generated fewer pressure surges reaching a maximum of ∼ 200 kPa and ∼ 350 kPa (Fig. 7b & c) respectively. The majority of the pressure magnitudes reached ∼ 100 kPa and ∼ 150 kPa (Fig. 7b & c, respectively) corresponding to the values recorded in Fig. 5b. However, the overall accumulation of pressure magnitude was larger, indicated by the thickness of the corresponding signal in the time-domain (Fig. 7d), due to the addition of the Hf transducer enhancing the previously documented “mist” formation and rapid bubble oscillation in DIW:EtOH and DIW:IPA. The presence of the numerous tiny cavitation bubbles activated by the Hf source under the large cavitation bubbly clouds in these cases indicates a higher cavitation activity as recently explained.^31^ This further corroborates with the significantly larger 1st and 2nd harmonics (from over imposed cavitation bubble emissions at resonance size) demonstrated by the DIW mixture spectra compared to pure DIW (Fig. 6), in agreement with the earlier results.35 The larger pressures for DIW:EtOH and DIW:IPA also correlate with the greater measured P_RMS_ pressures (Fig. 5a), in addition to the larger graphene yields as demonstrated in Fig. 2b and Table S3.

Upon activation of the sonotrode (inset 1), DIW:EtOH and DIW:IPA display a faster nucleation of cavitation bubbles under the sonotrode tip (Fig. 7b & c) compared to DIW (Fig. 7a). The build-up of cavitation intensity generates larger pressure spikes for all liquids (inset 2), whereby DIW:EtOH and DIW:IPA produce the largest cavitation zone (54 and 59 mm^2^ cross sectional area, respectively). However, DIW (26 mm^2^ cross sectional area) produced the largest SW pressures due to the proclivity of localised bubble cloud collapse and, hence, a lesser detrimental effect from cavitation shielding. The high vapour saturation pressures of EtOH and IPA (around 6 kPa) compared to DIW (2.3 kPa) and significantly lower surface tension facilitate the earlier cavitation threshold [34] with increased number of small bubbles providing more spots for efficient exfoliation [35]. Therefore, on the one hand we have the lesser magnitude of SWs. On the other hand, the enhanced cavitation zone, in conjunction with the tiny “mist” cavitation bubbles (smaller, spread-out cavitation which have been previously shown [25] to be beneficial for exfoliation due to their ability to penetrate in-between partially exfoliated graphite layers, producing greater overall cavitation intensity) leads to a higher yield of high-quality graphene (in line with Fig. 2b) [37]. This is also evident from the presence of a stabilised cavitation regime (>20 ms) where a larger cavitation zone (inset 3) is maintained for DIW:EtOH and DIW:IPA and promotes effective exposure of GP to cavitation activity. Consequently, this leads to a higher yield. It is worth noting that the yield percentage (∼6%) reported in the current work is a characteristic of ULPE-produced graphene, which is even higher than 0.1 %, 0.3 %, and 2–5 % reported in the literature [38], [39], [40]. The exposure to additional satellite bubbles is also portrayed (Fig. 7b, inset 3 and 2), which further aids in providing oscillating forces to shear graphite layers apart.

**Fig. 8:**
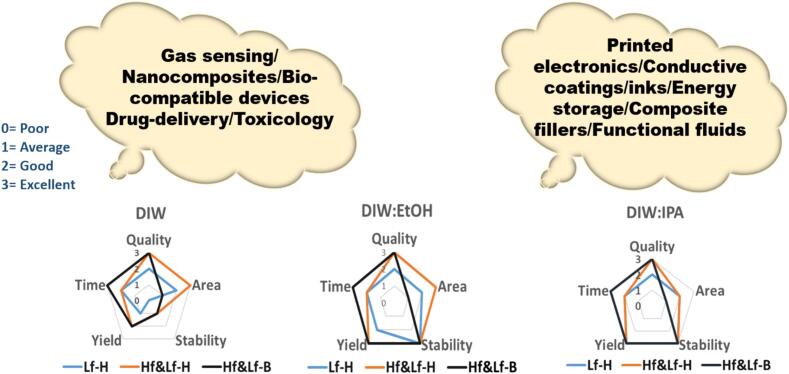
The evaluation of DIW, DIW:EtOH and DIW:IPA in terms of producing FLG yield, processing time, quality, area, stability and yield in the purposed Hf&Lf-H/B set-up for different areas of applications. Note for area: number indicates size differences in graphene flakes, lowest (1) and highest numbers (3) indicate small and large sized flakes respectively.

It is also worthy to discuss the credibility of DIW:EtOH and DIW:IPA over using EtOH and IPA as individual solvents. In one of our previous works, the low cavitation efficiency of ethanol [33], [34], [35] was demonstrated, resulting in smaller amount of graphene (∼0.01 mg/ml) produced [14]. The superior wettability, dispersibility, perfect surface tension matching with surface energy of graphite [41] explains the rationale behind why DIW:EtOH/DIW:IPA (note that IPA features physical and chemical properties in line with EtOH) are superior candidates over EtOH and IPA. Exfoliation efficiency of each of the studied solvents has been evaluated in terms of quality, size, yield and stability of FLG, suggesting some sort of flexibility of obtaining FLG with different lateral sizes and thicknesses, which finds its potential in wide spectrum of cutting-edge applications, presented in Fig. 8.

## 4 Conclusions

In this study, a novel dual-frequency reactor utilizing different sonotrode sizes to produce graphene in environmentally friendly solvents was deployed. The goal of this study was to comprehend how solvents affect the formation of cavitation and the dynamics of bubbles. Additionally, the produced eco-graphene was characterized based on yield, quality, stability, thickness, and size.

The characterization results of the graphene samples, supported by the bubble dynamics and acoustic pressure analysis, indicate that the use of DIW:IPA and DIW:EtOH represent efficient, eco-friendly solvents for producing high-quality FLG flakes with tailored thicknesses (4–10 Ls), area of flakes (0.3–1.5 μm^2^) (DIW:EtOH produces significantly larger flakes than DIW:IPA), good yield (∼6%) and stable suspensions lasting over six-months (∼70 %) which offers flexibility in a wide-range of applications.

The findings indicate that under the sonotrode tip, cavitation development in DIW produces a more confined cavitation cloud and in contrast to DIW:IPA and DIW:EtOH mixtures where both produce a much larger spatial distribution of cavitation bubbles, including additional, and larger in size satellite bubbles. Furthermore, both DIW:EtOH and DIW:IPA produce tiny “mist” cavitation bubbles which further aid in enhancing the cavitation zone, in addition to faciliting exfoliation of graphite. Further cavitation analysis revealed larger measured acoustic pressures for dual frequency setups, particularly with the addition of EtOH or IPA, corresponding to greater graphene yields.

The effect of the sonotrode diameter was also investigated, which further controls and affects the exfoliation of graphene. It is shown that the larger size sonotrode (twice the diameter) can reduce the processing times by half while maintaining the same quality and yield levels. The similar pressures for both liquids indicate that the bell (B) sonotrode produces comparable yields to the horn (H) due to its double emitting surface that enlarges the cavitation zone and increases the amount of shock waves. This in combination with the role of the solvents can further enhance the exfoliation efficiency of the cavitation zone.

Our study unveils novel insights into the production of stable graphene dispersions in sustainable green solvents using our original dual frequency ULPE set-up. This work paves the way for diverse applications in cutting-edge technologies, including eco-graphene-based stable conductive inks to develop low-temperature processed electrically conductive graphene-based pastes and inks replacing metal-based electrodes to be used in perovskite solar cells which will reduce manufacturing costs and improve device recyclability, functionalized eco-friendly graphene for water-splitting processes in hydrogen generation, gas sensors, composites and aqueous based bio-friendly graphene is an excellent candidate for targeted drug delivery where biocompatibility is the primary concern. The importance of achieving stable dispersions is pivotal for graphene's commercialization, where end-users increasingly demand high-quality dispersions with high-throughput capabilities and extended shelf lives.

## CRediT authorship contribution statement

**Amanpreet Kaur:** Writing – original draft, Visualization, Validation, Methodology, Investigation, Formal analysis, Data curation, Conceptualization. **Justin A. Morton:** Writing – original draft, Methodology, Investigation, Data curation. **Anastasia V. Tyurnina:** Writing – review & editing, Methodology, Investigation, Data curation. **Abhinav Priyadarshi:** Investigation, Data curation. **Morteza Ghorbani:** Writing – review & editing. **Jiawei Mi:** Writing – review & editing, Funding acquisition. **Kyriakos Porfyrakis:** Writing – review & editing, Funding acquisition. **Dmitry G. Eskin:** Writing – review & editing, Visualization, Resources, Project administration, Funding acquisition, Conceptualization. **Iakovos Tzanakis:** Writing – review & editing, Visualization, Supervision, Resources, Methodology, Funding acquisition, Data curation, Conceptualization.

## Declaration of competing interest

The authors declare that they have no known competing financial interests or personal relationships that could have appeared to influence the work reported in this paper.

## Data Availability

The data that supports the findings of this study are available upon request from the corresponding author.

## References

[1] Hernandez Y. (2008). High-yield production of graphene by liquid-phase exfoliation of graphite. Nat. Nanotechnol..

[2] Sinclair R.C., Suter J.L., Coveney P.V. (2019). Micromechanical exfoliation of graphene on the atomistic scale. Phys. Chem. Chem. Phys..

[3] Xu S., Zhang L., Wang B., Ruoff R.S. (2021). chemical vapor deposition of graphene on thin-metal films. Cell Rep. Phys. Sci..

[4] Yu H., Zhang B., Bulin C., Li R., Xing R. (2016). High-efficient synthesis of graphene oxide based on improved hummers method. Sci. Rep..

[5] Lavin-Lopez M.P., Valverde J.L., Sanchez-Silva L., Romero A. (2016). Solvent-based exfoliation via sonication of graphitic materials for graphene manufacture. Ind. Eng. Chem. Process Des. Dev..

[6] Carey T., Alhourani A., Tian R. (2022). Cyclic production of biocompatible few-layer graphene ink with in-line shear-mixing for inkjet-printed electrodes and Li-ion energy storage. npj 2D Mater. Appl..

[7] Htwe Y.Z.N., Mariatti M. (2022). Printed graphene and hybrid conductive inks for flexible, stretchable and wearable electronics: progress, opportunities, and challenges. J. Sci.: Adv. Mater. Devices.

[8] Y. Fu, et al. Graphene related materials for thermal management. 2D Mater. **7(1)**, 012001-012043 (2020). doi: 10.1088/2053-1583/ab48d9.

[9] Sagade A.A., Aria A.I., Edge S. (2017). Graphene-based nanolaminates as ultra-high permeation barriers. npj 2D Mater. Appl..

[10] Ochapski M.W. (2021). Li Intercalation into multilayer graphene with controlled defect densities. Carbon Trends.

[11] Dybowska-Sarapuk L. (2018). Efficient inkjet printing of graphene-based elements: influence of dispersing agent on ink viscosity. Nanomater..

[12] Johnson D.W., Dobson B.P., Coleman K.S. (2015). A Manufacturing perspective on graphene dispersions. Curr. Opin. Colloid Interface Sci..

[13] Byrne F.P. (2016). Tools and techniques for solvent selection: green solvent selection guides. Sustain. Chem. Processes.

[14] Ng K.L. (2023). Direct evidence of the exfoliation efficiency and graphene dispersibility of green solvents toward sustainable graphene production. ACS Sustain. Chem. Eng..

[15] Tasis D., Papagelis K., Spiliopoulos P., Galiotis C. (2013). Efficient exfoliation of graphene sheets in binary solvents. Mater. Lett..

[16] Halim U. (2013). A rational design of cosolvent exfoliation of layered materials by directly probing liquid-solid interaction. Nat. Commun..

[17] Cai X., Jiang Z., Zhang X., Zhang X. (2018). Effects of tip sonication parameters on liquid phase exfoliation of graphite into graphene nanoplatelets. Nano. Res. Lett..

[18] Gayathri S., Jayabal P., Kottaisamy M., Ramakrishnan V. (2014). Synthesis of few layer graphene by direct exfoliation of graphite and a Raman spectroscopic study. AIP Adv..

[19] Choi E.Y., Choi W.S., Lee Y.B., Noh Y.Y. (2011). Production of graphene by exfoliation of graphite in a volatile organic solvent. Nanotechnol..

[20] Tyurnina A.V. (2021). Environment friendly dual-frequency ultrasonic exfoliation of few-layer graphene. Carbon.

[21] Kaur A. (2022). Temperature as a key parameter for graphene sono-exfoliation in water. Ultrason. Sonochem..

[22] Morton J.A. (2023). An Eco-friendly solution for liquid phase exfoliation of graphite under optimised ultrasonication conditions. Carbon.

[23] Morton J.A. (2021). New insights into sono-exfoliation mechanisms of graphite: In situ high-speed imaging studies and acoustic measurements. Mat. Today.

[24] Khavari M., Priyadarshi A., Hurrell A., Pericleous K., Eskin D., Tzanakis I. (2021). Characterization of shock waves in power ultrasound. J. Fluid Mech..

[25] Backes C. (2016). Spectroscopic metrics allow in situ measurement of mean size and thickness of liquid-exfoliated few-layer graphene nanosheets. Nanoscale.

[26] Tyurnina A.V. (2023). Effects of green solvents and surfactants on the characteristics of few-layer graphene produced by dual-frequency ultrasonic liquid phase exfoliation technique. Carbon.

[27] Ferrari A.C. (2006). Raman spectrum of graphene and graphene layers. Phys Rev. Lett..

[28] Eckmann A. (2012). Probing the nature of defects in graphene by Raman spectroscopy. Nano Lett..

[29] Ferrari A.C., Basko D.M. (2013). Raman spectroscopy as a versatile tool for studying the properties of graphene. Nat. Nanotechnol..

[30] Khan U., O’Neill A., Porwal H., May P., Nawaz K., Coleman J.N. (2012). Size Selection of dispersed, exfoliated graphene flakes by controlled centrifugation. Carbon N Y.

[31] Morton J. (2023). Dual frequency ultrasonic cavitation in various liquids: High-Speed imaging and acoustic pressure measurements. Phys. Fluids..

[32] Qin L. (2022). Ultrafast synchrotron X-ray imaging and multiphysics modelling of liquid phase fatigue exfoliation of graphite under ultrasound. Carbon.

[33] Tzanakis I., Lebon G.S.B., Eskin D.G., Pericleous K.A. (2017). Characterizing the cavitation development and acoustic spectrum in various liquids. Ultrason. Sonochem..

[34] Tzanakis I., Hadfield M., Henshaw I. (2011). Observations of acoustically generated cavitation bubbles within typical fluids applied to a scroll expander lubrication system. Exp. Therm. Fluid Sci..

[35] Khavari M. (2023). Cavitation-induced shock wave behaviour in different liquids. Ultrason. Sonochem..

[36] Carter C.B., Williams D.B. (2016). Transmission electron microscopy: diffraction, imaging, and spectrometry. Springer.

[37] Tyurnina A.V. (2020). Ultrasonic exfoliation of graphene in water: a key parameter study. Carbon.

[38] Zhang X. (2010). Dispersion of graphene in ethanol using a simple solvent exchange method. Chem. Comm..

[39] Goni F., Chemelli A., Uhlig F. (2021). High-yield production of selected 2D materials by understanding their sonication-assisted liquid-phase exfoliation. Nanomaterials.

[40] Paton K.R. (2014). Scalable production of large quantities of defect-free few-layer graphene by shear exfoliation in liquids. Nat. Mat..

[41] Shen J. (2015). Liquid phase exfoliation of two-dimensional materials by directly probing and matching surface tension components. Nano Lett..

[42] U. Khan, A. O’Neill, M. Lotya, S. De, J.N. Coleman. High-concentration solvent exfoliation of graphene. Small, **6(7),** (2010), 864–871 (2010). doi: 10.1002/smll.200902066. 10.1002/smll.200902066 20209652

[43] Telkhozhayeva M. (2021). Higher ultrasonic frequency liquid phase exfoliation leads to larger and monolayer to few-layer flakes of 2D layered materials. Langmuir.

[44] Chabot V., Kim B., Sloper B., Tzoganakis C., Yu A. (2013). High yield production and purification of few layer graphene by Gum Arabic assisted physical sonication. Scientific Reports.

