# Supplementary material for: Dual frequency ultrasonic liquid phase exfoliation method for the production of few layer graphene in green solvents
Source: Ultrason Sonochem. 2024 Jun 14;108:106954. doi: 10.1016/j.ultsonch.2024.106954 (PMC11211887; doi:10.1016/j.ultsonch.2024.106954)
Supplement: Supplementary Data 1 [file mmc1.docx]

**Supplementary**

**Dual frequency ultrasonic liquid phase exfoliation method for the production of few layer graphene in green solvents**

Amanpreet Kaur^1*^, Justin A. Morton^1, 2^, **Anastasia V. Tyurnina^3^,**  Abhinav Priyadarshi^1^, Morteza Ghorbani ^4^ , Jiawei Mi^5^, Kyriakos Porfyrakis^6^, Dmitry G. Eskin^3^ & Iakovos Tzanakis*^1,7^

^1^Faculty of Technology, Design and Environment, Oxford Brookes University, Headington, Oxford, OX3 0BP

Wheatley, Oxford, OX33 1HX, UK

^2^Department of Engineering Science, University of Oxford, Parks Road, Oxford,

OX1 3PJ, UK

^3^Brunel Centre for Advanced Solidification Technology, Brunel University London, Kingston Lane, London, UB8 3PH, UK

^4^Faculty of Engineering and Natural Science, Sabanci University, 34956 Tuzla, Istanbul, Turkey

^5^Department of Engineering, University of Hull, Cottingham Rd, Hull, HU6 7RX, UK

^6^Faculty of Engineering and Science, University of Greenwich, Central Avenue, Chatham Maritime, Kent, ME4 4TB, UK

^7^Department of Materials, University of Oxford, Parks Road, Oxford, OX1 3PH, UK

**^*^**E-mail of corresponding author: [itzanakis@brookes.ac.uk](mailto:itzanakis@brookes.ac.uk), akaur@brookes.ac.uk

**1.1 The optimization of input transducer powers required for efficient exfoliation based on matching of peak-to-peak amplitude of horn (H) (22 mm) and bell (B) sonotrodes (40 mm)**

The series of ULPE experiments were conducted to investigate the optimum input powers required for efficient graphene exfoliation in Hf&Lf configuration. To achieve this, peak-to-peak amplitudes of both H and B sonotrodes were matched for two different input transducer powers. In the first case (Figure S1a, b), ULPE was performed for 20% input power (28±2W) with H and 50% input power (100±2W) with B sonotrode keeping peak-to-peak amplitude constant i.e. 9 μm. In the second case (Figure S1c, d), ULPE was performed for 40% input power (68±2W) with H and 100% input power (224±2W) with B sonotrode keeping constant peak-to-peak amplitude i.e. 18 μm. Subsequently, UV-Vis spectra of resultant supernatants (approx. 5 ml) extracted during ULPE after a certain time intervals were recorded to monitor the progress of exfoliation. Figure S1 presents the representative UV-Vis spectra for the collected series of supernatants. The rationale behind choosing DIW:EtOH as opposed to DIW can be found from our previous work.^1^ From Figure S1a, broad peaks centred on 272-300 nm for 20% input power (H) were noticed, indicating obvious graphitic signals (despite 120 min sonication) highlighted in dotted region. On the contrary in Figure S1b, distinct characteristics features of graphene centred on ~266 nm, attributed to π-π* transitions of aromatic C=C bonds have been detected, with the prominent and intense peaks registered for 60 min, demonstrating the enhanced degree of exfoliation, dispersion-uniformity and the likelihood of few layer graphene (FLG) flakes.^2^ We understand that for 20% input power with H sonotrode, delivered power was not adequate to break the large graphite crystallites completely, which makes the case for inefficient exfoliation, whereas 50% input power with B sonotrode, delivered power (as indicated in Figure S1b) was comparatively higher, which is surplus for efficient exfoliation. From Figure S1c,d, characteristics peaks were observed with the registry of broad graphitic peaks ~ 275 nm at 30 min, which further shifts towards 266 nm with the progression of time. On the contrary, no appreciable graphene related peaks (highlighted in dotted region in Figure S1d) were found in samples processed with 100% input power (B). The absence of graphene peaks can be explained on the basis of enormous delivered power due to which cavitation shielding^3-4^ played an important role in impeding the continuous transmission of shock waves (SWs) far away from the sonotrode, which in turn suppresses the shearing of graphite.


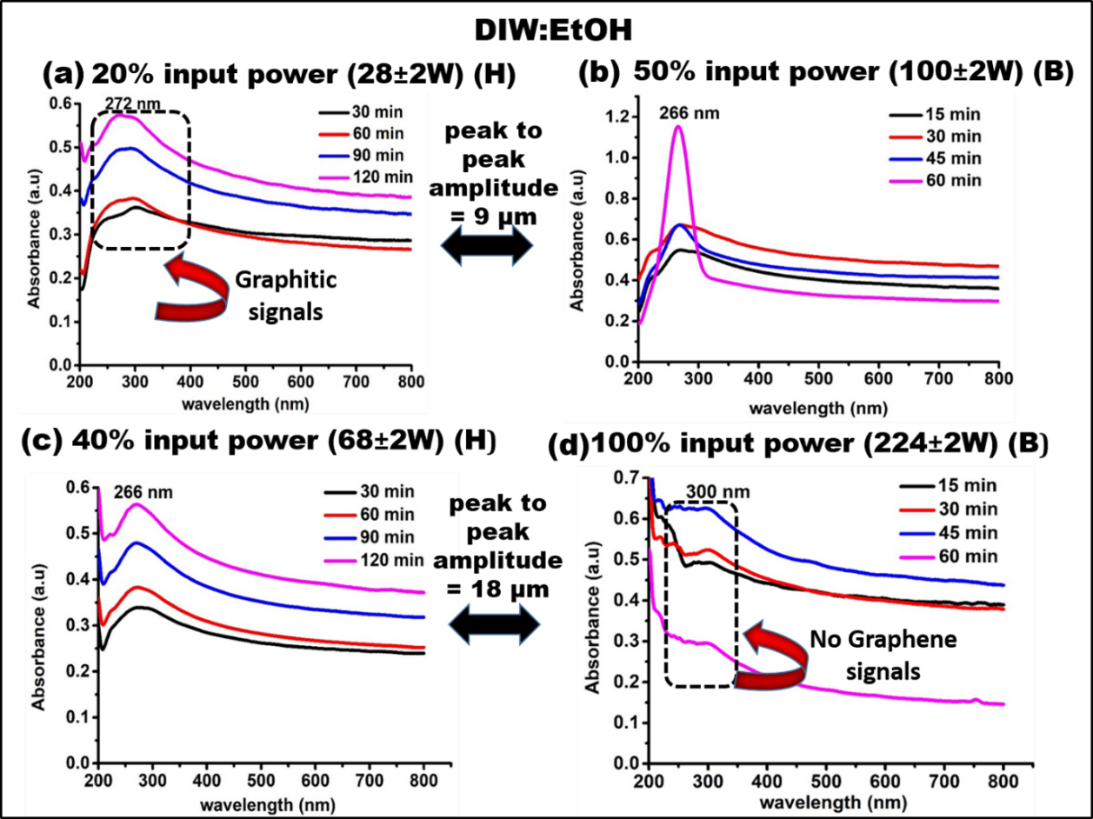


**Figure S1 (a-d)** UV-Vis spectra of obtained supernatants after series of ULPE in DIW:EtOH corresponding to peak-to-peak amplitudes of 9 μm and 18 μm. H & B refer to horn and bell sonotrodes.


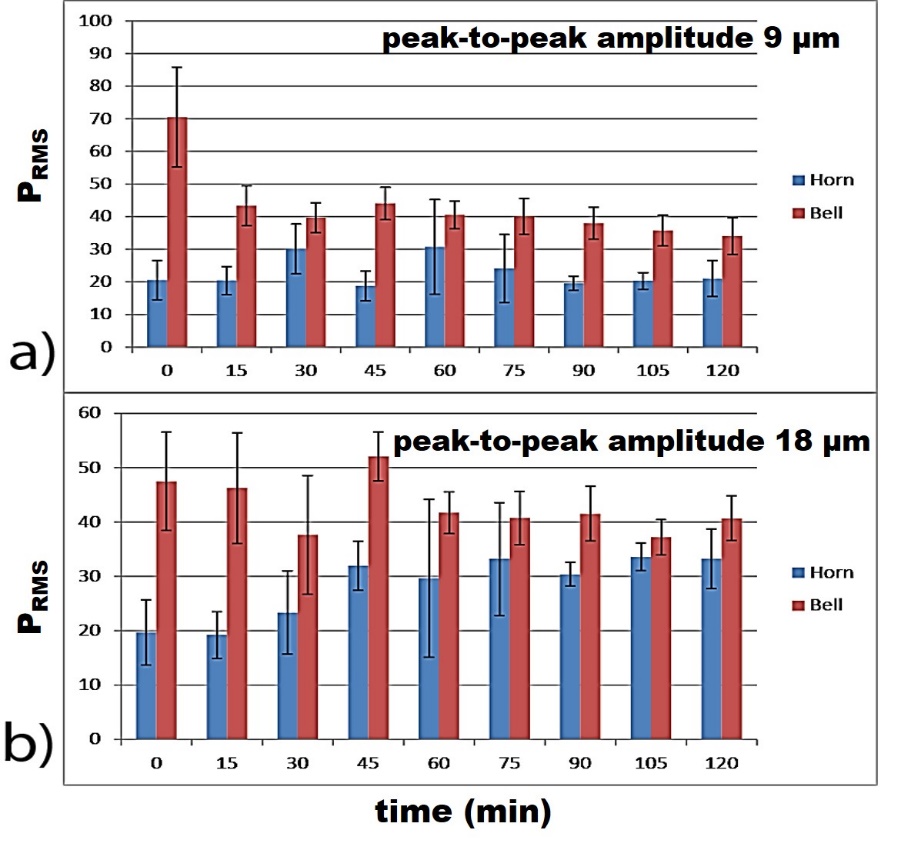


**Figure S2** Acoustic pressures for the H and B sonotrodes using a) 20% and 50% input power, respectively (9 µm peak-to-peak amplitude; b) 40% and 100% input power, respectively (18 µm peak-to-peak amplitude).

Concurrently with results given in Figure S1, acoustic pressures were recorded during ULPE, in intervals of 15 minutes to monitor the exfoliation progress (Figure S2). This was conducted for two input power scenarios; 20% and 50% input power for the H and B sonotrodes, respectively (Figure S2a), and 40% and 100% input power for the H and B sonotrodes, respectively (Figure S2b).

The first scenario demonstrated the B sonotrode (50% input power) to have approximately twice the pressures as the H (Figure S2a). That`s why well-defined graphene peaks were identified in Figure S1b. On the other hand, with both input powers doubled (Figure S2b) we saw the bell sonotrode to only generate marginally larger pressures than in Figure S2a, indicating a lack of efficiency, likely caused by cavitation shielding due to the larger bubbly clouds formed under the sonotrode tip. These larger pressures (corresponding to 100% input power) may also increase the likelihood of damaging effects to the exfoliated nanosheets, which correlates well with Figure S1d. It is to be noted that 40% or 50% input power setting using H sonotrode gives similar acoustic pressure measurements and power delivered to the solution. Additionally, from the obtained results in Figure S1-2 and our previous reported works^1, 9^ on using 50% power with H sonotrode, the optimized input power for all ULPE experiments in main manuscript was set at 50% power for both the H and B sonotrode to increase exfoliation efficiency and reduce acoustic shielding. In the case of the H sonotrode acoustic pressures rose, increasing with sonication duration. At later stages of ULPE, the horn pressures are of similar magnitude to the bell sonotrode, hence, providing sufficient pressures for exfoliation (larger cavitation cloud and more frequent SW generation), in addition to producing a more appropriate acoustic flow to recirculate graphitic particulates into the cavitation zone. The following figures (Figure S3-6) display various preliminary ULPE tests at different input powers.


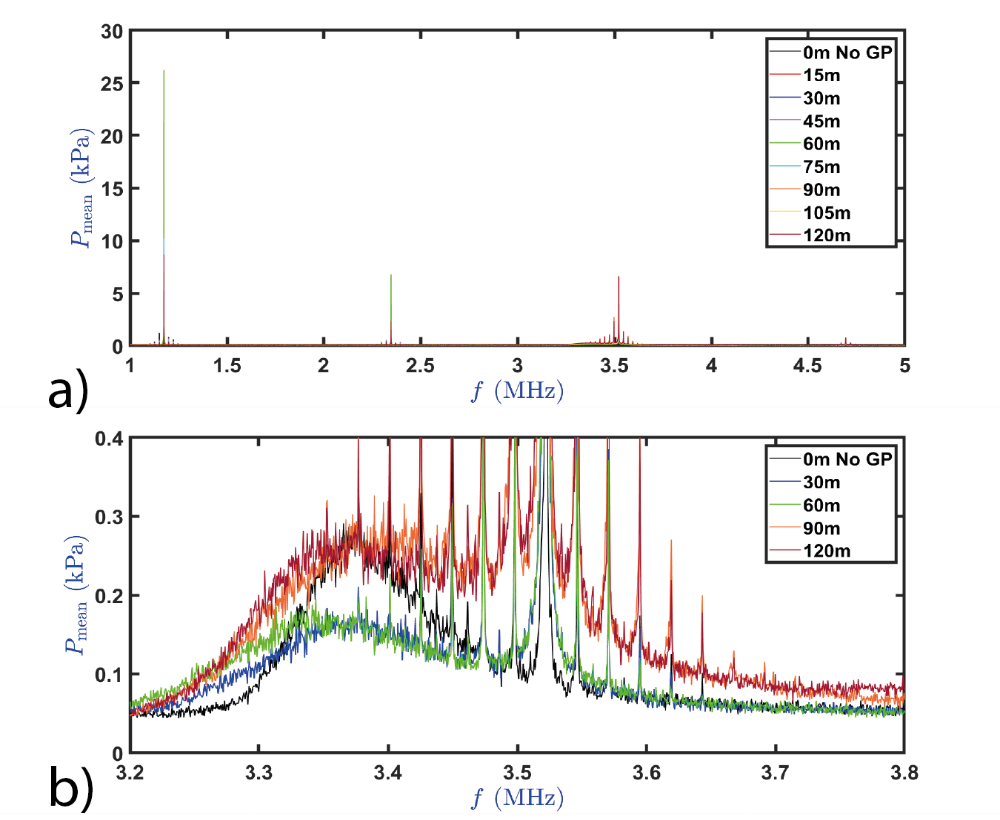


**Figure S3** a) Acoustic spectra and b) SW peak development over 120 minutes using the H sonotrode at 20% input power (9 µm peak-to-peak amplitude). Note that some time intervals are removed from SW peak view to prevent crowding of data points.

Acoustic spectra for the H sonotrode at 20% input power gave rise to a most prominent 1^st^ harmonic, with the 2^nd^ and 3^rd^ about 3.5 times lesser in magnitude (Figure S3a). Analysis of the SW emissions represented by the hump^1,4^ in Figure S3 demonstrated a general increasing in magnitude with sonication duration, indicating the process of exfoliation (unimpeded SW fronts making contact with the fibre optic hydrophone as larger graphite particles (GP) are broken down) (Figure S3b).


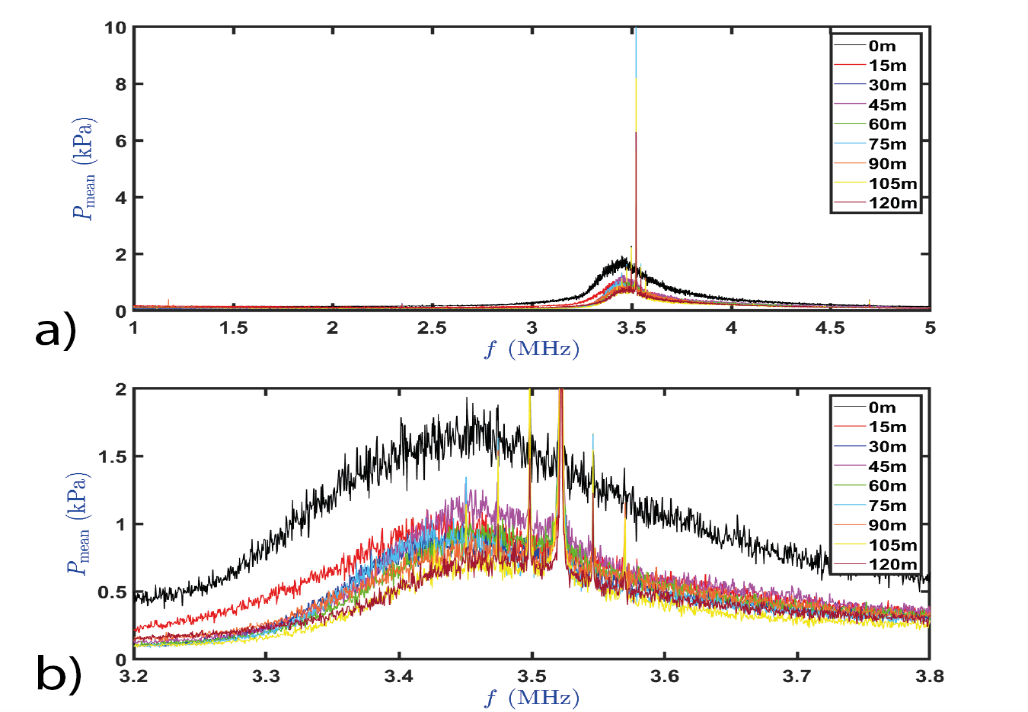


**Figure S4** a) Acoustic spectra and b) SW peak development over 120 minutes using the B sonotrode at 50% input power (9 µm peak-to-peak amplitude).

Acoustic spectra for the B sonotrode at 50% input power gave rise to a most prominent 3^rd^ harmonic, with the 1^st^ and 2^nd^ almost entirely suppressed. The overall pressure is significantly lower than the H sonotrode (Figure S4a), whereas the SW hump is greater in magnitude (Figure S4b) (as discussed in the main manuscript). In this instance, the SW peaks are mostly clustered together during the time intervals of the ULPE duration.


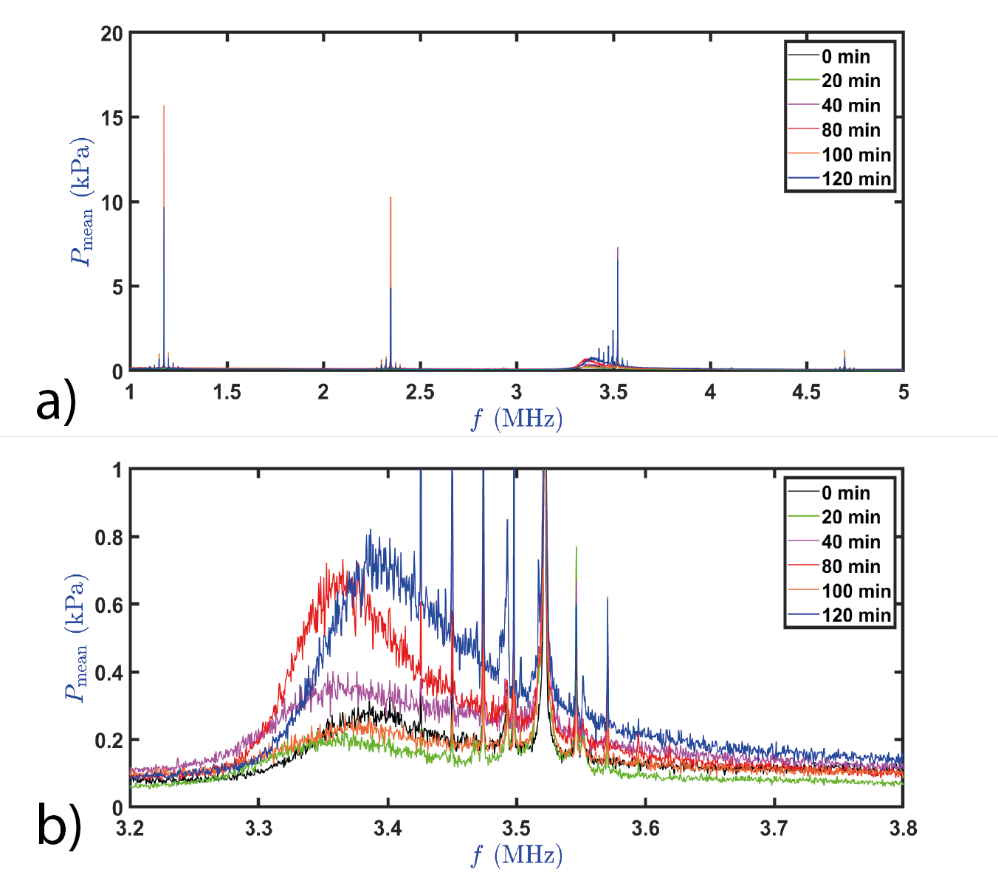


**Figure S5** a) Acoustic spectra and b) SW peak development over 120 minutes using the H sonotrode at 40% input power (18 µm peak-to-peak amplitude).

Acoustic spectra for the H sonotrode at 40% input power demonstrated the 1^st^ harmonic to be most prominent, with the 2^nd^ and 3^rd^ descending in magnitude (Figure S5a). Later periods of ULPE were shown to produce harmonics of greater magnitude. Analysis of the SW hump again demonstrated a general increasing in magnitude with sonication duration, indicating exfoliation progression (Figure S5b).


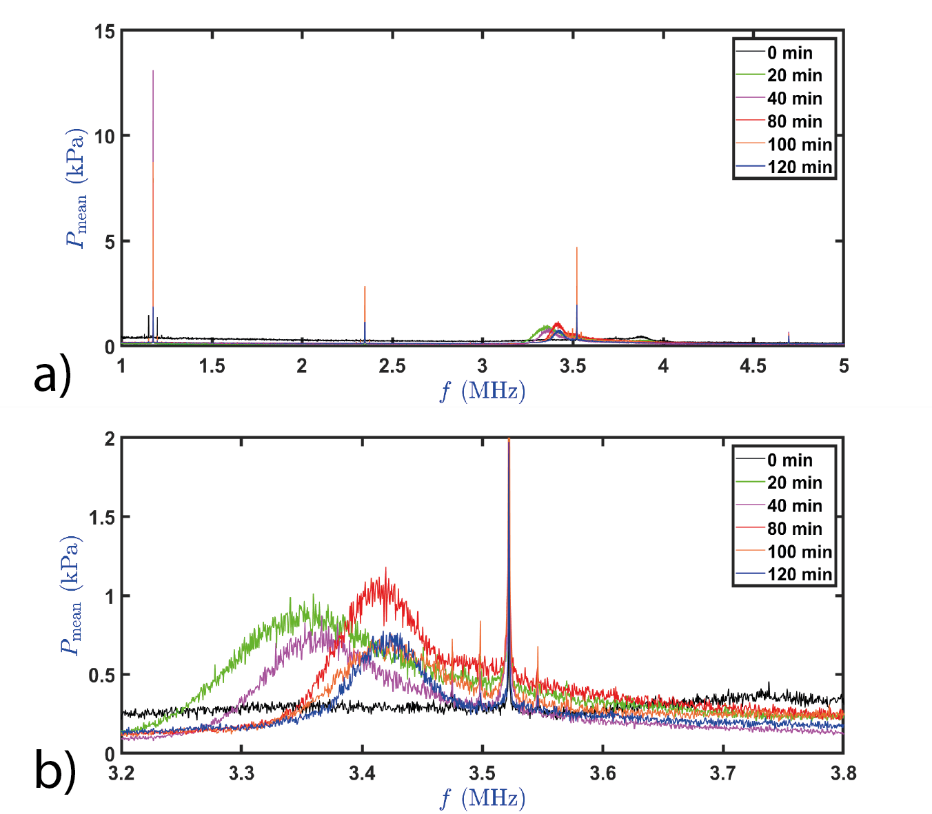


**Figure S6** a) Acoustic spectra and b) SW peak development over 120 minutes using the B sonotrode at 100% input power (18 µm peak-to-peak amplitude).

Using the B sonotrode at 100% input power gave rise to a most prominent 1^st^ harmonic (Figure S6a). The overall pressures were closer in magnitude to the H sonotrode in this instance. As ULPE continued, the 3^rd^ harmonic increased in magnitude (as seen in Figure S6b), which correlates with Figure 6b in the Main manuscript (whereby the spectra were taken in pure water/ethanol and hence closer to an exfoliated graphite solution). The SW hump in this instance generally tended to increase in magnitude with sonication duration (0.25 to 1 kPa), as well as sharpening of the humps (Figure S6b). Interestingly, as the duration of ULPE increased, the SW hump up-shifted to larger frequencies (in most instances). This phenomenon is likely due to the unimpeded SW propagation as graphite particulates are exfoliated, hence, the speed of the SWs is also unimpeded, giving rise to this frequency increase.


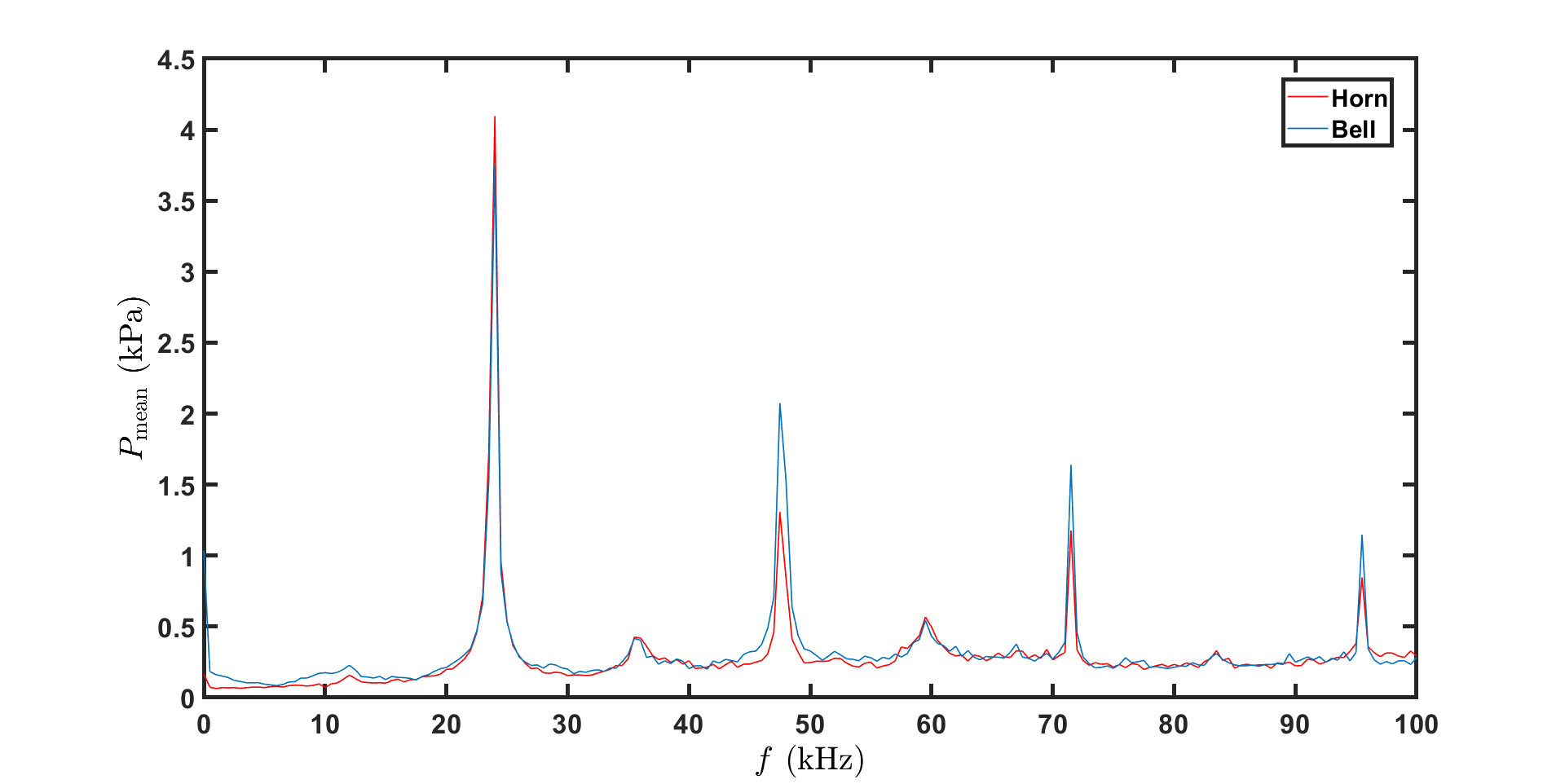


**Figure S7** Example acoustic spectra of the LF range (0-100 kHz) for both the H and B sonotrode in DIW.

Figure S7 shows a qualitative spectra of both the H and B in the LF range up to 100 kHz. The 1^st^ resonant frequency is shown to be more dominant for the H sonotrode, whereas the 2^nd^ to 4^th^ harmonics display the B sonotrodes dominance (with increases in the range of 20 to 40%).

- 1. **The effect of sonication time on ULPE of graphene in DIW, DIW:EtOH and DIW:IPA at dual frequency i.e. Hf (1174 kHz) from bottom and Lf (24 kHz) from top with both Horn (22 mm) and Bell sonotrode (40 mm) at 50% optimized input power, 40±5^o^C.**

The additional experiments were carried out to monitor the progress of exfoliation at the optimized input powers i.e. 50% (discussed in previous section 1.1). Figure S8 (a-f) demonstrates the representative UV-Vis spectra of resultant supernatants obtained during ULPE after a certain time interval to monitor the progress of exfoliation. It is to be noted that H samples were observed from 30-120 min, whereas 15-60 min for B samples. We interpret the previously mentioned observation as B sonotrode having double diameter (40 mm) to that of H sonotrode (22 mm) with obvious larger cavitation zone (Figure S9), which requires less threshold time to exfoliate graphene completely. The motive behind choosing 15-60 min is power delivered to the solution is approximately double using B sonotrode (as indicated in Figure S1) and extended cavitation zone produced by B sonotrode (Figure S9) and it is discussed further and demonstrated in Figure S10a, b. From the spectra (Figure S8a-f), the absorption peaks related to graphene centred at 266-267 nm had been noticed1 in each case. For the first instance, intensity of absorption peaks (266-267 nm) increases with sonication time, which is understandable that exfoliation is progressing with time. The maximum absorption intensity values (corresponding to 266-267 nm) (indicated with black squares) and qualitative estimate of concentration i.e. A/ℓ (indicated with red circles) as per Lambert–Beer’s law, A/ℓ (at 660 nm) = αC (where A is measured absorbance, ℓ is optical path length, α is extinction coefficient and C is concentration of dispersion) had been extracted^2^ and plotted in Figure S8 (g-l). From the plots, it is evident that linear increase in concentration of produced graphene for each solvent has been perceived in which nearly saturation state can be seen after 90 min for H samples (Figure S8 (g,i, k)) which was also the case for Lf-ULPE processed samples from our previous reports.^1^ On the other hand, there is a continuous increase in concentration of graphene produced with B sonotrode. From A/ℓ values, it is interesting to mention here that concentration of produced graphene with B sonotrode in 60 min is approximately equivalent to the produced with H sonotrode in 120 min. Besides, declining values of ratios of Full Width Half Maximum to Abs (max) (FWHM/Abs (max)) provided in inset of Figure S8 (g-l) manifest progress of graphene exfoliation with sonication time.

*
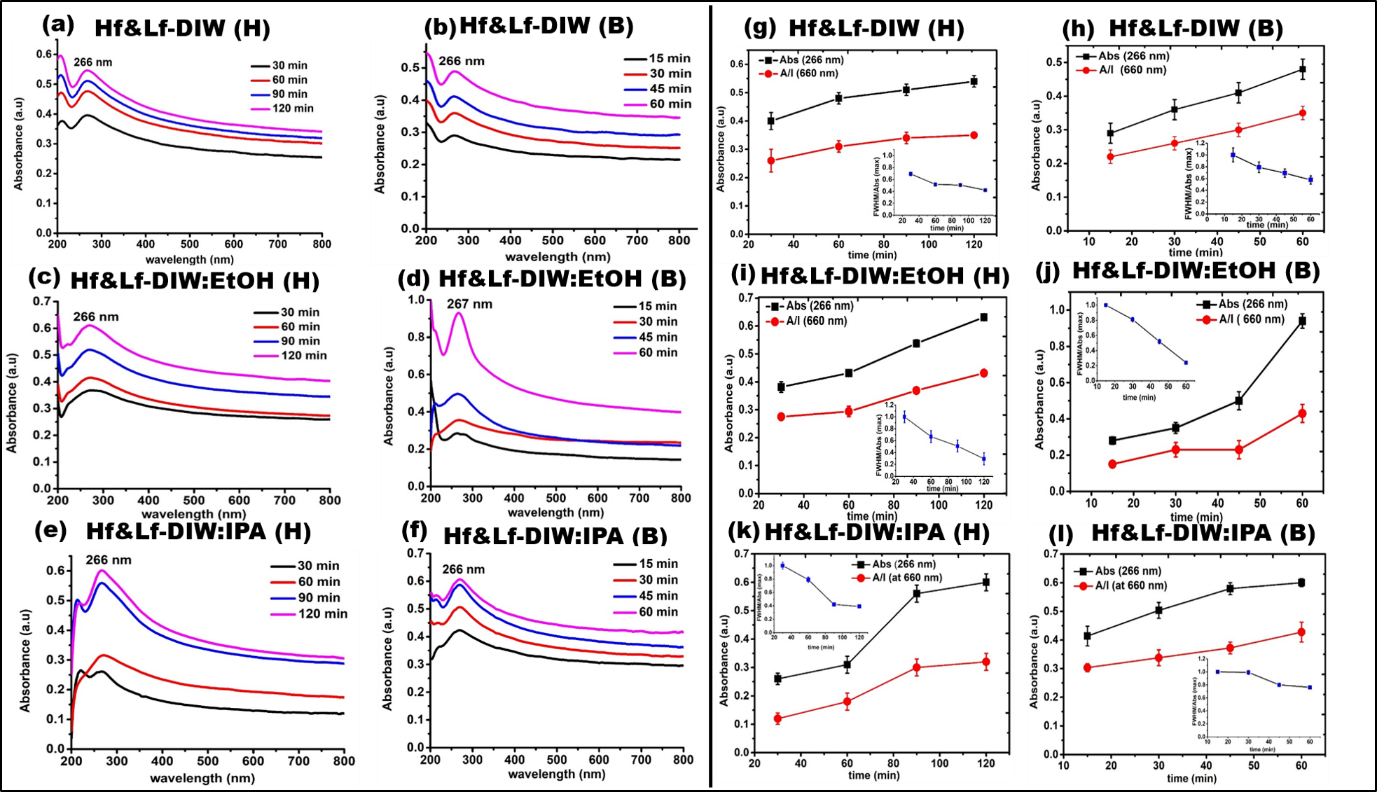
*

**Figure S8** (a-f) Representative UV-Vis spectra of obtained supernatants as a function of sonication time for both H and B sonotrode; (g)-(l) plots feature the trend of Abs (266 nm) and A/ℓ (660 nm) as a function of sonication time in DIW, DIW:EtOH and DIW:IPA.


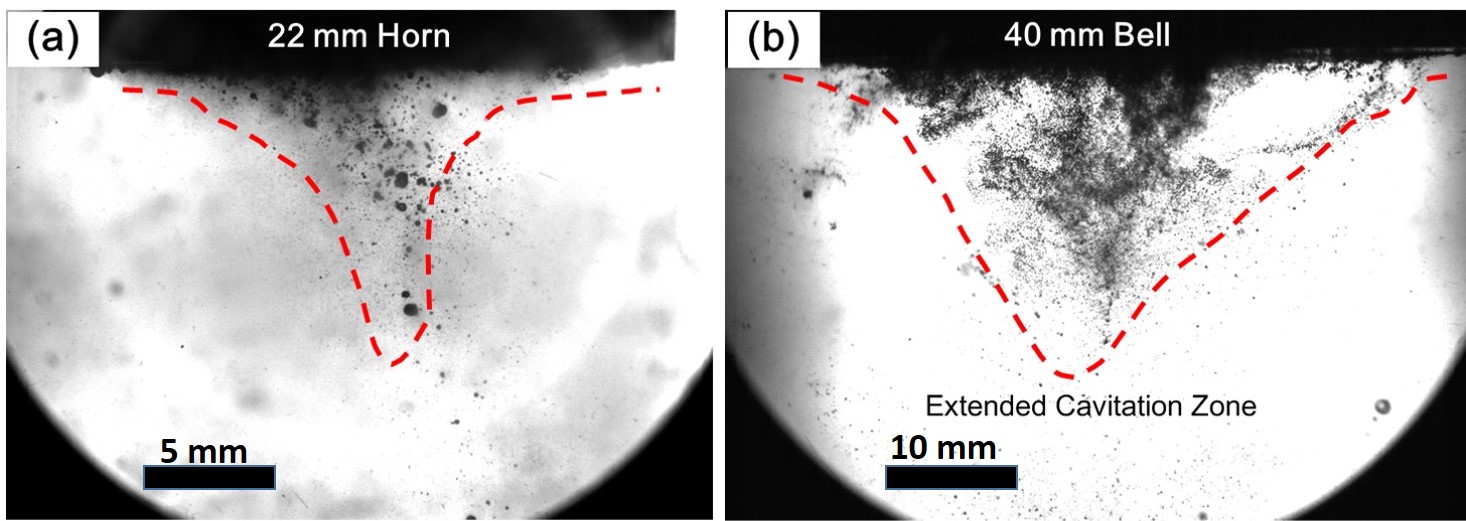


**Figure S9** Cavitation zone comparison with a) 22 mm H sonotrode b) 40 mm B sonotrode.

In order to probe the end point for the achievement of exfoliation as a function of time, an experiment was conducted in which approx. 5 ml dispersions were pipetted out during the course of sonication (Hf&Lf, 50% input power, 40±5^o^C in DIW:EtOH with B sonotrode) after every 15 min up to 120 min and centrifuged to obtain the supernatants. The resultant supernatants were tested for UV-Vis absorption studies and their normalized absorption spectra are given in Figure S10a. The magnified image of spectra is provided in its inset of Figure S10a. From figure, we can see that in first 30 min, graphitic peaks corresponding to 298-300 nm were obtained, which further blue-shifted from 278 nm (45 min) to 269 nm (60 min onwards), indicative of successful progression of exfoliation with time explicitly after 60 min. Figure S10b demonstrates saturation in concentration of graphene after 60 min (highlighted with grey shaded region). We interpret the results based on typical images of extended cavitation zone cavitation zone (Figure S9) of obvious double area produced by bell in contrast to horn, which leads to emission of more SWs, which are driving mechanism of exfoliation.^3^ Therefore, effective treatment time reduced to half with larger cavitation zone as more GP particles circulate into treatment volume and exfoliated which makes the case for efficient exfoliation in half duration.

Furthermore, indistinguishable concentration trend has been registered during ULPE in DIW by keeping constant input power for both horn and bell sonotrode (Figure S10c), signifying H and B sonotrode can perform identically at same input transducer powers.

Based on the outcomes of the results, we have identified the best suitable processing parameters for graphene exfoliation in DIW, DIW:EtOH and DIW:IPA. Further advanced characterization studies (provided in Main manuscript file) were carried out for the samples processed at optimized parameters of temperature 40 ^o^C (previous work),^9^ input power 50% (section 1.1), sonication time 2 h and 1 h for H and B sonotrodes (section 1.2) respectively.


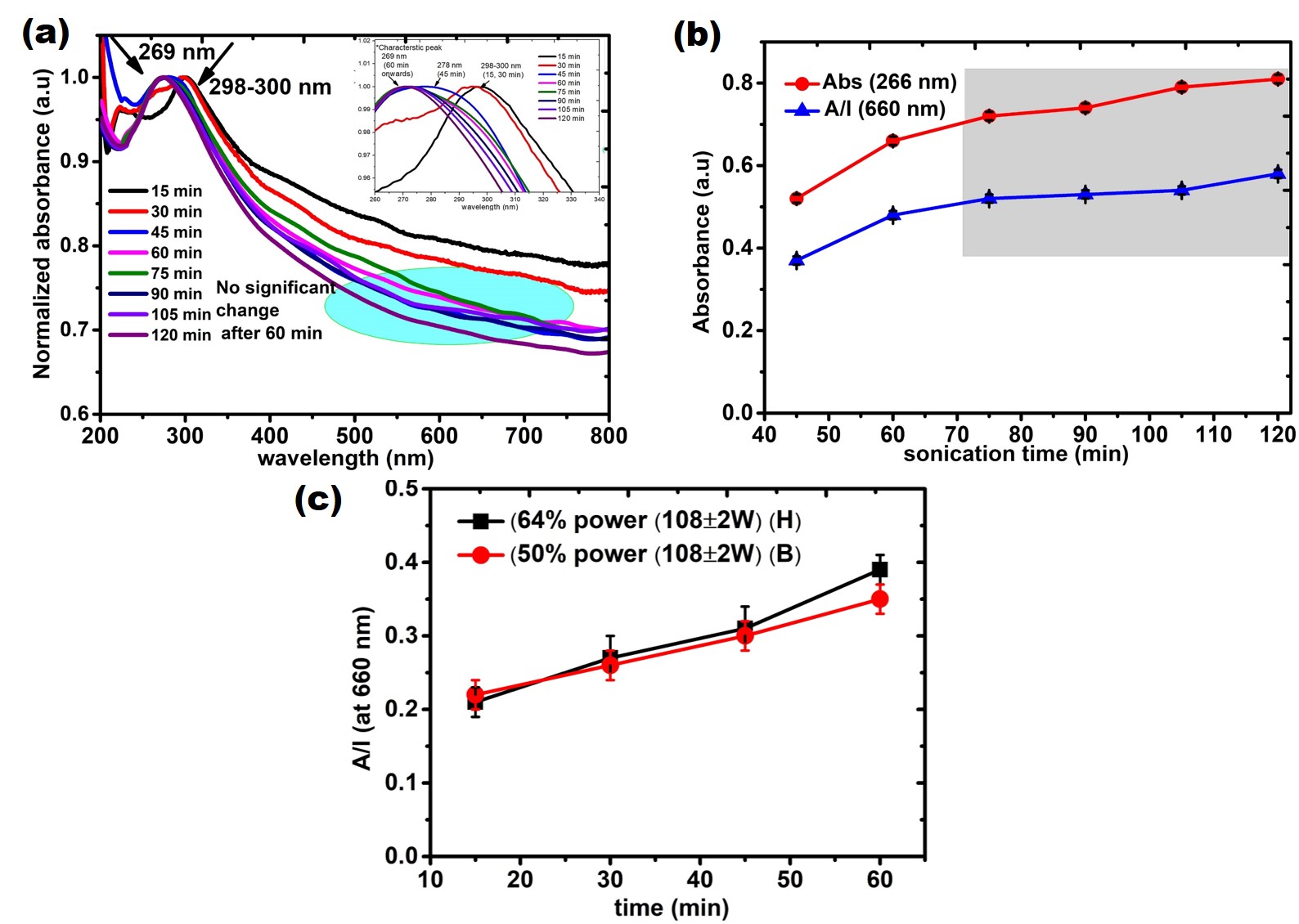


**Figure S10** (a) Normalized absorption spectra recorded as a function of sonication time (using B sonotrode); inset shows magnified image of peak shifting with time; (b) plot of Abs (max) and A/ℓ (at 660 nm) as a function of sonication time; the saturation regime is highlighted with the grey shaded region; (c) plot highlights A/l (at 660 nm) with sonication time at constant input powers.

To understand the exfoliation efficiency of solvent under examination, graphite flake (GF) (99% carbon, Sigma Aldrich 100 mesh, 149 μm) was put under ultra-sonication treatment in DIW, DIW:EtOH and DIW:IPA at constant 50% input power which is 92±2W, 85±2W and 82±2W respectively. It should be noted that power consumption in DIW:EtOH/DIW:IPA is less in comparison to DIW and as expected (see physical properties Table S2) which can be perceived as an advantage in terms of a reduced threshold and energy consumption required to exfoliate graphene in DIW:EtOH/DIW:IPA on top of the other benefits that these green solvents offer in the process as explained in the main manuscript. The reason behind choosing GF over GP for conducting this experiment is to visualise clearly the macro-exfoliation/ fragmentation slicing of GF post sonication (Figure S11). It was observed during the experiment that GF completely broke into smaller graphite particulates in DIW:EtOH and DIW:IPA within 1 min (Figure S11b, c), whereas large sized GF remain suspended on the liquid surface in DIW even for 5 min sonication (Figure S11a). The size differences under microscope (Keyence digital VHX) can be noticed in Figure S11a-c. Another test was conducted to sonicate GF in DIW for 1 min at maximum 100% input power (180±2W) to compare with 1 min sonication in DIW:EtOH and DIW:IPA (From Figure S11b, c), nevertheless, GF did not break completely which can be seen from Figure S11d. These experimental observations are consistent with molecular dynamic (MD) simulations performed by Chen *et al.*^5^ From their MD simulations, it has been established that different solvents have different starting forces (or shear forces) to peel off the graphene from graphite, specifically demonstrating the largest starting force required for graphene exfoliation in water as opposed to alcohols/mixes.


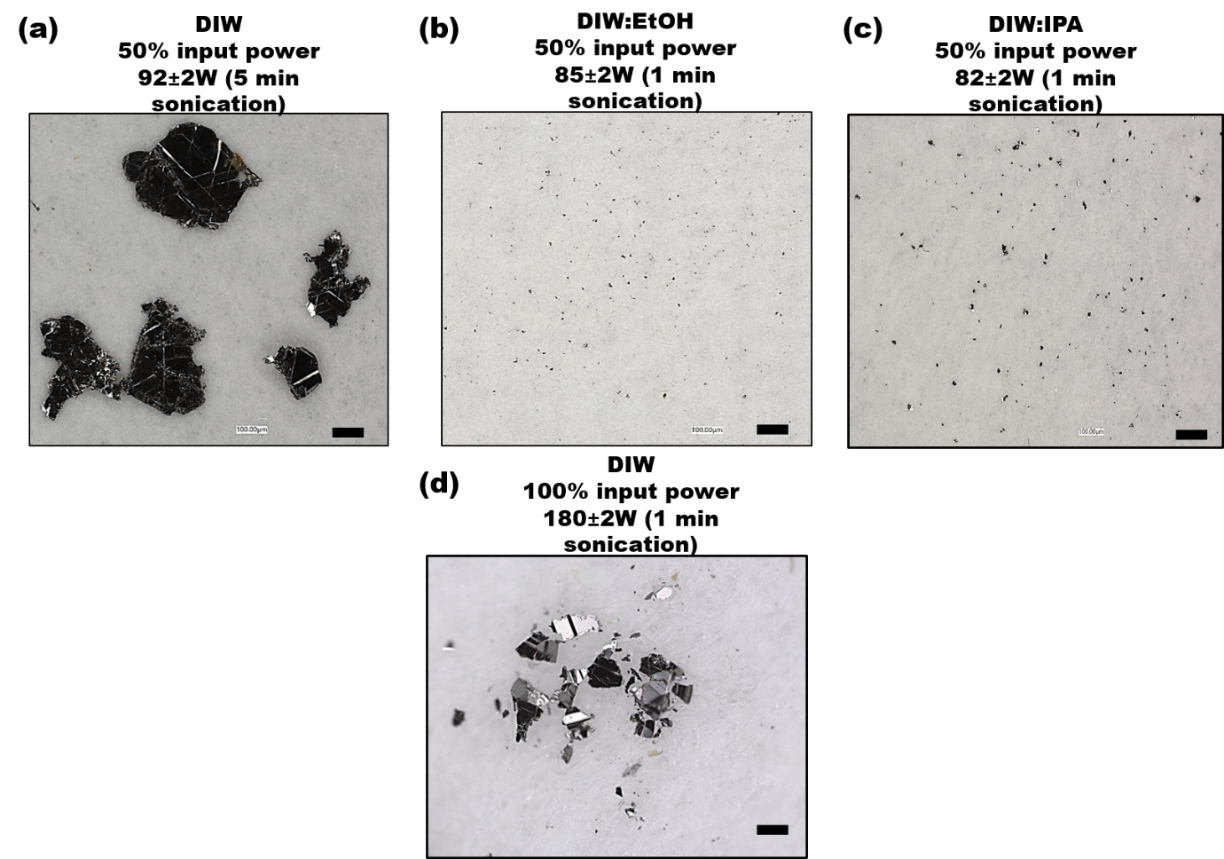


**Figure S11** (a-d) Microscopic images of graphite flakes after sonication; evaluation of exfoliation efficiencies of each investigated solvent, DIW, DIW:EtOH and DIW:IPA. (Scale bar is 100 μm).

Furthermore, Shen *et al.*^6^ provided a “Surface Tension Components Matching theory” (STCM) to exfoliate targeted 2D materials in a high yield. Efficient solvents for ULPE were found to be those which have closer values of ratio of polar to dispersive components (σ^p^/ σ^d^ ) of surface tension to the 2D materials in question. Likewise, STCM theory explains both exfoliation efficiency and stabilization effects. From theory, if σ^p^/σ^d^ of cosolvent mixtures (which is DIW:EtOH and DIW:IPA in present study) matches well to that of targeted 2D material (GP in our case), then exfoliation ability, dispersibility and stability can be significantly improved. From table S2, DIW:EtOH/DIW:IPA (50:50) are found to be the best matching σ^p^/ σ^d^ value (0.46/0.48) with bulk graphite (0.47) whilst having significant (about 50% less) with graphite`s total surface tension ($\sigma= \sigma p+\sigma d)$. Interestingly, one can see from table S2, DIW:EtOH and DIW:IPA have close matching σ^p^/ σ^d^ values with NMP, DMF and DMA solvents (commonly used solvents for exfoliation), which makes robust case for the replacement of toxic solvents with eco-friendly alcohol based co-solvents. Following from the results of Figure 2(a), (b) and table S2, it is emphasized that σ^p^/σ^d^ is the most relevant parameter for explaining the improved exfoliation efficiency, dispersibility and stability of graphene in both DIW:EtOH and DIW:IPA in contrast to DIW.

Apart from σ^p^/ σ^d^, the improved yield/stability of graphene in DIW:EtOH and DIW:IPA can be elucidated on the basis of long hydrocarbon chains of attached -CH_3_ groups in alcohol-water co-solvent molecules, which reduces the Leonard-Jones forces of attraction (L-J attraction) between the sheets due to their larger steric repulsion which eventually lessens restacking of graphene layers. In other words, high viscosity owing to larger molecular weight of co-solvent molecules prolongs the stability of suspended graphene flakes for a long time as the movements of flakes become slower in high viscous mediums (see table S2). Therefore, aforesaid arguments seem conducive to our observations explaining enhanced exfoliation ability, yield and stability of graphene in DIW:EtOH and DIW:IPA in contrast to DIW.

**Table S1** The enlisted values of acoustic intensity (W/m^2^) and sonication energy (kJ/ml)

in DIW, DIW:EtOH and DIW:IPA in Hf&Lf configuration using H and B sonotrodes.

| Solvent/  Sonotrode | P_liquid_-P_air_^*^ (W) | ^#^Acoustic Intensity (W/m^2^)×10^4^ | ^&^Sonication energy, E (kJ/ml) |
| --- | --- | --- | --- |
| DIW  Horn  Bell | 233±2W  283±2W | 61.50  23 | 11.18  13.58 |
| DIW:EtOH  Horn  Bell | 223±2W  273±2W | 58.84  21.73 | 10.70  13.10 |
| DIW:IPA  Horn  Bell | 220±2W  268±2W | 58.04  21.33 | 10.56  12.86 |
|  |  |  |  |

^#^Using the formula; acoustic intensity= $\frac{P_{\mathrm{liquid}}-P_{\mathrm{air}} \left( W \right)}{Area of sonotrode \left( m^{2} \right)}$

*Where $P_{\mathrm{liquid}}$ is the acoustic pressure measured in the solution, $P_{\mathrm{air}}$ is the pressure measured when the sonotrode was activated in the atmosphere, $P_{\mathrm{air}} \left( Horn \right)=17W$; $P_{\mathrm{air}} \left( Bell \right)=$ 14W; corresponding to 50% input power, peak to peak amplitude, 23 μm and 9 μm for horn and bell sonotrode respectively.

$Area of sonotrode$ 3.79×10^-4^ m^2^ (horn sonotrode, 22 mm diameter); 12.56×10^-4^ m^2^ (bell sonotrode, 40 mm diameter). ^&^Sonication energy = $\frac{(P_{\mathrm{liquid}}-P_{\mathrm{air}} \left( W \right))\times sonication time (s)}{Volume of solution(ml)}$ ; volume of liquid =150 ml; sonication time = 2 h (7200 s).

**Table S2** Physical properties of different solvents i.e. surface tension^6^, σ^p^/ σ^d7^ and viscosity^8^ of different solvents at 20^o^C.

| Solvents | Surface tension (mN m^-1^)  σ | Polar component (mN m^-1^)  (σ^p^) | Dispersive component  (mN m^-1^)  (σ^d^) | Polar/dispersive component  σ^p^/ σ^d^ | Viscosity  (mPa.s) |
| --- | --- | --- | --- | --- | --- |
| DIW | 72.75 | 50.65 | 22.10 | 2.292 | 1.00 |
| DIW:EtOH (50:50) | 28.51 | 7.31 | 15.91 | **0.460** | 1.28 |
| DIW:IPA (50:50) | 24.78 | 8.17 | 16.96 | **0.482** | 1.65 |
| NMP | 40.79 | 11.58 | 29.21 | **0.396** | 1.29 |
| DMF | 36.50 | 11.30 | 25.20 | **0.448** | 1.89 |
| DMA | 38.04 | 11.49 | 26.55 | 0.433 | 0.94 |
| Ethanol | 22.31 | 4.40 | 19.30 | 0.228 | 1.18 |
| Propanol | 21.74 | 2.80 | 21.90 | 0.128 | 2.37 |
| Graphite | 50.71 |  |  | **0.471** |  |

**Table S3** The quantitative data for all the investigated parameters of obtained FLG flakes in the studied solvents.

| **Sonotrode** | **Solvent** | **Input power (50%)**  **(W)** | **Time**  **(h)** | **P_RMS_**  **_(kPa)_** | **P_MAX_**  **_(kPa)_** | **D/G** | **2D/G** | **Intensity ratios^#^**  **D/D’** | | **Area (μm)^2^** | **NLs** | **Y^*^%**  **(±0.5)** | **Stability^@^**  **(%)** |
| --- | --- | --- | --- | --- | --- | --- | --- | --- | --- | --- | --- | --- | --- |
| Hf&Lf-H  (22 mm) | DIW | 250  ±2 | 2 | 32±1.8 | 196±20 | 0.29±0.07 | 0.95±0.13 | 1.88±0.5 | 1.5  ±0.5 | | 4±1 | 3.75 | 38 |
|  | DIW:  EtOH | 240  ±2 | 2 | 39±6 | 165±6 | 0.34±0.12 | 0.87±0.2 | 1.75±0.34 | 1.4  ±0.8 | | 10±5 | 5.80 | 69 |
|  | DIW:  IPA | 238  ±2 | 2 | 35±4 | 151±7 | 0.39±0.09 | 1.32±0.55 | 1.5±0.1 | 0.7  ±0.5 | | 8±4 | 6.5 | 72 |
|  |  |  |  |  |  |  |  |  |  | |  |  |  |
| Hf&Lf-B  (40 mm) | DIW | 265  ±2 | 1 | 32±0.7 | 115±9 | 0.3±0.06 | 0.99±0.21 | 1.49±0.15 | 0.2  ±0.06 | | 6±1 | 3.0 | 30 |
|  | DIW:  EtOH | 255  ±2 | 1 | 34±1 | 104±9 | 0.31±0.08 | 1±0.16 | 1.5±0.1 | 0.27  ±0.24 | | 6±3 | 5.75 | 70 |
|  | DIW:  IPA | 250  ±2 | 1 | 32±5 | 122±5 | 0.36±0.08 | 1.32±0.55 | 1.5±0.1 | 0.35  ±0.2 | | 5±2 | 5.5 | 69 |
|  |  |  |  |  |  |  |  |  |  | |  |  |  |
| Lf-H  (22 mm) | ^$^DIW | 92  ±2 | 2 | 27±0.5 | 171±15 | 0.55±0.31 | 0.65±0.19 | 2.55±0.32 | 0.5  ±0.4 | | 5±2 | 1.25 | 8 |
|  | ^$^DIW:EtOH | 82  ±2 | 2 | 20±1.3 | 96±5 | 0.37±0.02 | 0.63±0.18 | 2.24±0.17 | 1.1  ±1.0 | | 4±2 | 3.75 | 78 |
|  | DIW:  IPA | 80  ±2 | 2 | 15±1 | 91±12 | 0.43±0.13 | 0.84±0.2 | 1.86±0.39 | 0.7  ±0.2 | | 7±1 | 5.8 | 75 |

# derived from Raman data; * sensitive to centrifugation speed and initial concentration of GP; @ after <180 days; $ data from our previous work^1^

**References**

[1] Morton, J. et al. An eco-friendly solution for liquid phase exfoliation of graphite under optimised ultrasonication conditions. *Carbon* N Y **204**, 434-446 (2023). [https://doi.org/10.1016/j.carbon.2022.12.f](https://doi.org/10.1016/j.carbon.2022.12.070)

[2] Backes, C. et al. Spectroscopic metrics allow in situ measurement of mean size and thickness of liquid-exfoliated few-layer graphene nanosheets. *Nanoscale* **8(7)**, 4311-4323 (2016). <https://doi.org/10.1039/c5nr08047a>

[3] Morton, J. et al. New insights into sono-exfoliation mechanisms of graphite: In situ high-speed imaging studies and acoustic measurements. *Mat. Today* **49**, 10-22 (2021). <https://doi.org/10.1016/j.mattod.2021.05.005>

[4] Khavari, M., Priyadarshi, A., Hurrell, A., Pericleous, K., Eskin, D., & Tzanakis, I. Characterization of shock waves in power ultrasound. *J. Fluid Mech.* **915**, 1-14 (2021). <https://doi.org/10.1017/jfm.2021.186>

[5] Chen, S., Li, Q., He, D., Liu, Y., Wang, L., & Wang, M. Dynamic exfoliation of graphene in various Solvents: All-atom molecular simulations. *Chem. Phys. Lett.* **804**, 139900 (2022). <https://doi.org/10.1016/j.cplett.2022.139900>

[6] Shen, J.et al. Surface tension components based selection of cosolvents for efficient liquid phase exfoliation of 2D materials. *Small* **12(20),** 2741-2749 (2016).  <https://doi.org/10.1002/smll.201503834>

[7] Halim, U. et al. A rational design of cosolvent exfoliation of layered materials by directly probing liquid-solid interaction. *Nat. Commun.* **4,** 3213 (2013). DOI: 10.1038/ncomms3213

[8] Alam, M. S., Ashokkumar, B., & Siddiq, A. M. The density, dynamic viscosity and kinematic viscosity of protic polar solvents (pure and mixed systems) Studies: A theoretical insight of thermophysical properties. *J. Mol. Liq.* **251**, 458-469 (2018). <https://doi.org/10.1016/j.molliq.2017.12.089>

[9] Kaur, A. et al. Temperature as a Key Parameter for Graphene Sono-exfoliation in Water. *Ultrason. Sonochem.* **90,** 106187 (2022). <https://doi.org/10.1016/j.ultsonch.2022.106187>
